# LIP formation and protracted lower mantle upwelling induced by rifting and delamination

**DOI:** 10.1038/s41598-018-34194-0

**Published:** 2018-11-08

**Authors:** Kenni Dinesen Petersen, Christian Schiffer, Thorsten Nagel

**Affiliations:** 1 Department of Geoscience, Aarhus University, 8000 Aarhus, Denmark; 2 Department of Earth Sciences, Durham University, Durham, DH1 3LE UK

## Abstract

Large Igneous Provinces (LIPs) are commonly attributed to mantle plumes, hot upwellings from the deep lower mantle, apparently unrelated to plate motions. However, LIPs often form in association with rifting and breakup. Using numerical modelling, we introduce a novel idea that explains plume-like mantle upwelling by plate tectonic processes. Our model indicates that rifting-induced delamination of orogenic lithosphere can perturb the thermochemical mantle stratification and induce lower mantle upwelling which causes syn-rift LIP formation followed by protracted and enhanced mid ocean ridge basalt (MORB) generation. Our model provides an explanation for the geographical correlation between the Caledonian suture, the North Atlantic Igneous Province (NAIP) and present-day Icelandic magmatism.

## Introduction

Continental breakup between North America and Europe in the late Palaeocene was accompanied by the formation of the NAIP, one of the classic LIPs. More than 1.3 × 10^6^ km^2^ of the present day North Atlantic region were covered by up to 1 × 10^7^ km^3^ of flood basalts^1,2^. Magmatism occurred in pulses between 62 and 54 Ma with a major outburst within the continental rift system that eventually became the North Atlantic Ocean^2,3^. Following continental breakup, the North Atlantic remained magmatically hyperactive, and thick (possibly >30 km) igneous crust was produced along the Greenland-Iceland-Faeroe Ridge (GIFR)^1^. Present-day Iceland similarly represents a mid-ocean ridge with anomalous topography, melt productivity and composition^2,4–6^.

The concept of mantle plumes, active upwellings with distinct chemical characteristics sired in the deep lower mantle^7^ explains many first-order properties of the NAIP as well as other LIPs^3,8^. High melt productivity, trace element and isotopic compositions of melts reflect elevated mantle potential temperatures (T_p_) and fertile melt sources, possibly with components of recycled crust^5^ and primordial mantle^6,8^. Finally, low velocity anomalies in seismic tomography reaching into the lower mantle have been interpreted as plume conduits^9^. These observations are widely seen as evidence for plumes originating from the deep lower mantle independently of plate motion. However, most LIPs formed in the context of continental rifting and breakup^10^ and are thus causally related to plate tectonic processes. The NAIP developed just where a Caledonian suture was dissected by Palaeogene rifting and breakup^11,12^, and the unlikeliness of a plume coinciding with exactly this intersection has been invoked as an argument against a deep origin^13^. Alternative ‘top-down’ explanations for LIP generation include edge-driven convection^14^, recycling of subducted oceanic crust^15^ and delamination of lithospheric mantle^16^. However, these non-plume models have difficulties explaining the particular chemical signature of melts and the long-lived high melt productivity that prevails today in Iceland 55 Myr after initial LIP formation^17^.

We hypothesise that orogenic lithosphere with a dense crustal root in eclogite facies^18^ may delaminate upon rifting^19^, rapidly sink through the upper mantle, penetrate the lower-upper mantle boundary (LUMB), and perturb the metastable stratification across this thermochemical boundary layer^20–22^. This would induce plume-like upwelling from the hot lower mantle and thus produce voluminous magmatism.

**Figure 1:**
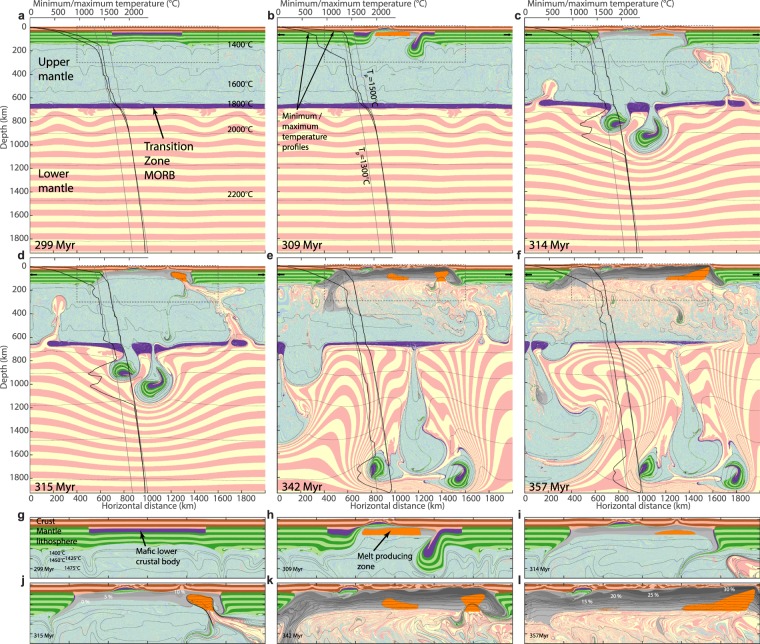
Snapshots of six stages of numerical modelling evolution. Cross sections showing state of numerical model 1 Myr prior to the onset of extension (**a**), and 9, 14, 15, 42 and 57 Myr after, respectively (**b**–**f**). Colour striping is used to discriminate between crust (red/tan), mantle lithosphere (light/dark green), upper mantle (light blue/tea green) and lower mantle (cream/pink), and to show strain since model onset. Purple indicates mafic material, orange indicates regions were melts are currently forming, and grey tones with white text show total degree of melt depletion of mantle material that melted at earlier stages of the model run. Thin black lines are isotherms, and bold black lines are minimum and maximum temperature as functions of depth. For comparison, grey curves show theoretical adiabats for T_p_'s of 1300 and 1500 °C, respectively. (**g**–**l**) close ups of central crustal region (dashed boxes in **a**–**f**).

To explore this hypothesis, we use 2D-thermo-mechanical modelling. Our model accounts for pressure- and temperature dependent viscous, elastic and plastic flow in the lithosphere-mantle system^23,24^, and for decompression mantle melting. Relevant physical properties associated with mineralogical changes are tracked using tabulated thermodynamic, free energy minimised models of density, heat capacity and entropy (methods). The modelling domain is 2000 × 2000 km (Fig. 1, Supplementary Figure S1) and encompasses the upper and lower mantle. Initially, a 600 km wide orogen comprising thickened continental crust with 20 km of mafic lower crust is placed in the centre of the model. A 200 °C T_p_ step is assumed across the LUMB, and a 40 km thick basaltic layer is placed below the mantle transition zone representing a MORB graveyard^25^ which is neutrally buoyant at this depth^26^ (Supplementary Figures S1 and 2).

**Figure 2:**
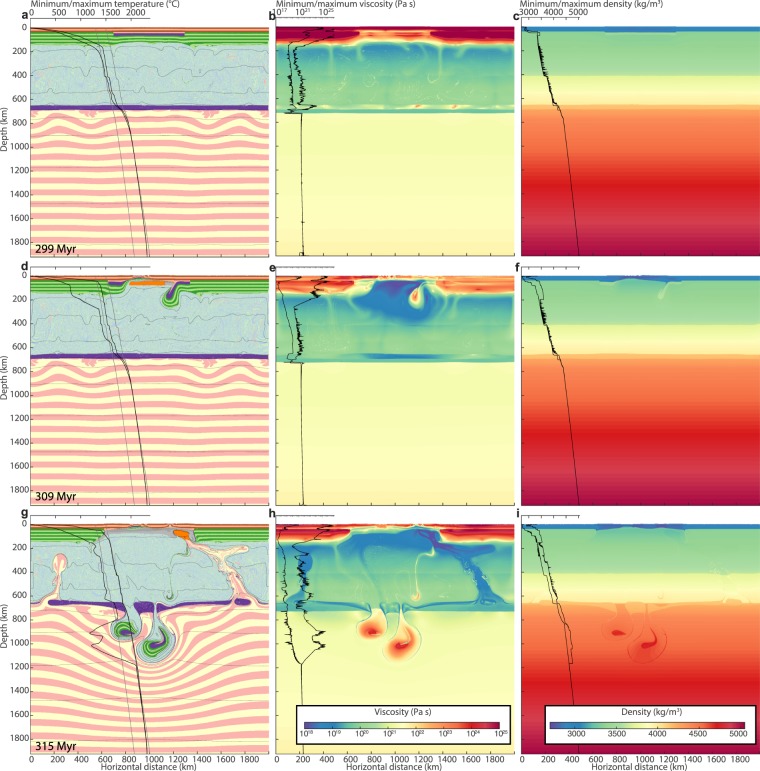
Viscosity and density structure. First row shows lithology (**a**), viscosity (**b**) and density structure (**c**) 1 Myr prior to the onset of extension. Colors and labels for (**a**) are as in Fig. 1a. Black lines in (**b**) and (**c**) show profiles of minimum and maximum values of viscosity and density and viscosity, respectively, as a function of depth. Viscosity is calculated as half the ratio of a second invariant of the deviatoric stress tensor, $\sigma {^{\prime} }_{II}=\sqrt{\sigma {^{\prime} }_{kk}}$, to a second invariant of the deviatoric strain rate tensor, $\dot{{\epsilon }}{^{\prime} }_{II}=\sqrt{\,\dot{{\epsilon }}{^{\prime} }_{kk}}$. (**d**–**f**) show the same properties for 9 Myr after the onset of extension. (**g**–**i**) show the same properties for 15 Myr after the onset of extension.

In order to test the metastable nature of the heavy orogenic root and the thermal boundary layer at the LUMB, tectonic quiescence with a velocity field of 0 across vertical model boundaries is assumed during the first 300 Myr of the simulation. Vigorous and continuous convection immediately develops in the upper mantle, while the lower mantle and the lithosphere remain stagnant. The mechanical separation between the upper and lower mantle due to phase transitions and compositional stratification^25,26^ persists, and the lower mantle retains an elevated T_p_ (Fig. 1a,g). Also, the partly eclogitised and negatively buoyant lower crust in the orogen remains metastable against delamination due to high strength of the cold mantle lithosphere (Fig. 2a–c).

**Figure 3:**
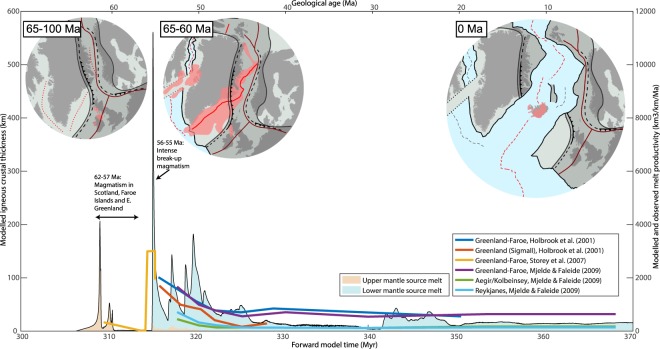
Model melt productivity and source compared to NAIP magmatic evolution. Black curve enclosing pink or blue area below shows model time (lower x-axis) vs. melt productivity and source (upper and lower mantle, respectively) of the model shown in Fig. 1. Thick, coloured curves show age (upper x-axis) of Cenozoic melt productivity estimates in the vicinity of present-day Iceland^1,2,4^. A model time of 370.5 Myr is assumed to correspond to the present (0 Ma) for comparison with the NAIP. Circular insets show plate reconstructions with locations of rift zones, approximate outlines of major magmatic activity and locations of Caledonian structures. Methods and sources for the data presented in the figure are described in the methods section.

At 300 Myr, extension is applied and the orogenic lithosphere begins to thin. During the first 9 Myr of extension, strain localises in a 200 km wide rift zone with a lithospheric thinning-factor of 2-3. Lithosphere thinning is associated with increasing horizontal density gradients between the lithosphere and asthenosphere, promoting convective instabilities proximal to the rift^27^. These instabilities are enhanced by the presence of the dense eclogitic lower crust. During further extension, abrupt delamination of the lithospheric mantle from the crust^16,19^ occurs as two separate drips within 2 Myr. The resulting asthenospheric counterflow causes a short-lived pulse of decompression melting (Figs 1b,h, 2d–f and 3).

Within 2 Myr, the delaminated lithospheric bodies sink through the upper mantle, reach the MTZ and penetrate the LUMB. This perturbs the metastable thermomechanical boundary layer and hot, buoyant lower mantle material rises to the rift zone causing voluminous melting (Figs 1c,d,i,j and 2f,g). The time between delamination and the onset of decompression melting of lower mantle upwellings is 6 Myr, and within 0.3 Myr, melt productivity (here defined as the volume of melt that is generated in a given time step normalised by extension rate; see also methods) reaches 280 km, 40 times average mid ocean ridge productivity^28^ (Fig. 3). The high productivity is a consequence of both a large upwards flux of lower mantle material and its high T_p_.

Lower mantle upwelling is caused by the delaminating material that sinks into the lower mantle and shears the MORB graveyard-layer so that it locally thickens towards regions of downwelling and thins further away. Where the MORB layer is thinned, Rayleigh-Taylor instabilities of buoyant lower mantle material form and start penetrating into the upper mantle. The MORB graveyard-layer, that hitherto had suppressed convective mixing between upper and lower mantle^29^ for 300 Myr, is thereafter breached as the lower mantle convective instabilities continue to grow, and as the delaminated material continues to sink into the lower mantle while dragging a wake of cold upper mantle material along (Fig. 1c–e). Plume-like lower mantle upwelling continues to increase melt productivity for the 20 Myr that follow the arrival of the first upwelling and melting of lower mantle material, and average igneous crustal thickness reaches ~35 km, with peaks 5–10 times higher (Fig. 3). The high productivity is, apart from a higher lower mantle temperature and a fast upwelling rate, also a consequence of the thinned lithosphere (due to rifting and delamination), allowing for a relatively shallow upper limit of the melt of the melt-forming region of ~35 km, close to the base of the continental crust (Fig. 1j,k). The transient variability of melt productivity is a consequence of asthenospheric small-scale convention that modulates the upwelling flow from the lower mantle and pulsation of lower mantle upwelling.

At 330–340 Myr, melt productivity is relatively low (5–10 km), because lower mantle upwelling slows down at this time and is not entrained into the rift zone. From ca. 342 Myr, a new pulse of lower mantle upwelling arrives into the rift system and increases melt productivity to 20–40 km for the next 10 Myr (Figs 1e,k and 3). In the following, pulsing and ascending hot lower mantle material continues to flow towards the thinned lithosphere, but gradually the sublithospheric, originally upper mantle, material becomes replaced with hot lower mantle material to depths greater than the maximum depth of melting at ~160 km (Fig. 1f,l). Consequently, decompression melting of a hot lower mantle source continues, but flow within the melt zone is, in turn, predominantly driven by the passive flow associated with plate separation, rather than buoyantly enhanced active upwelling (Fig. 1d,j). The elevated and stable T_p_ in the melt-generating zone leads to an average crust productivity of 16 km at times later than 50 Myr after the onset of extension.

Our model implies that plume-like upwelling of lower mantle material can be a direct cause of plate tectonic processes. It provides an explanation of why LIPs develop just where rifting intersects orogenic lithosphere and sutures. We propose that the pre-LIP magmatism in the NAIP at ca. 61–55 Ma is a consequence of rifting and delamination, while the intense activity during and following LIP formation at ~55 Ma was caused by actively rising lower mantle following a breach of the LUMB. This is consistent with models that point towards elevated temperatures and enriched or primordial melt source^5,6,8^. Also, low seismic velocities^9^, a high geoid and elevated topography^30^ would be consistent with our model. Our model predicts magmatic pulsation due to chaotic interaction between active upwelling and upper mantle convection. We suggest that such upper mantle modulation could explain observations that have been attributed to plume pulsation^4,31^.

The initially elevated lower mantle T_p_, assumed in our model, is a possible consequence of extended periods of stagnation where the lower mantle would gradually heat up due internal radiogenic heating and/or conductive heat flow from the core^32^. Different estimates of radiogenic heat production would correspond to heating rates up to 160 to K per Gyr, ignoring heat loss and heating from the core (see supplementary note on radiogenic heating rate).

Large low shear velocity provinces at the core-mantle boundary have been suggested to correlate with historical positions of LIP formation, and it has been hypothesised that they control the generation of plumes and LIPs^33^. Another explanation involves the effect of thermal insulation beneath supercontinents^34^. Our model of a top-down control on lower mantle upwelling does not preclude the presence of either these mechanisms. Instead, it provides a triggering mechanism for rapid tapping and destabilisation of a regionally heated lower mantle reservoir. Such destabilisation would likely manifest as plume-like upwelling of various magnitude or as a regional mantle overturn, depending on the amount of trapped heat and its distribution. The latter would also determine the duration of upwelling, as the process of delamination-induced flow from the lower mantle would be limited by the amount of hot lower mantle available. The generic model presented here assumes that the whole lower mantle is uniformly hot and upwelling therefore continues until the end of the simulation at 100 Myr after the onset of extension (Fig. 3). The development of an improved model with a melt productivity evolution more consistent to that of the NAIP, or other LIPs, would require knowledge on the specific mechanisms that heat the lower mantle and its thermal state prior to the onset of extension.

The model presented here requires a process that maintains thermal stratification with an elevated T_p_ of the lower mantle relative to the upper mantle for extended periods of time. It has been shown in earlier works^21,22^ that, depending on the somewhat controversial^35^ magnitude of the negative Clapeyron slope of the Ringwoodite-out reaction near the LUMB, mantle convection is possibly layered due to the convection-inhibiting effect of this phase transition. For the thermodynamic database employed in the present study^36^, the Clapeyron slope is half that of several recent estimates, and the associated convection-inhibiting effect is therefore relatively small (supplementary discussion). However, the possible presence of a MORB layer at the LUMB^25,26^, as assumed here, additionally to sustain mantle stratification^29^ (compare with the model presented in Supplementary Figure S7 where no such MORB is assumed).

An additional vital component of the model presented here is the presence of a dense orogenic root that facilitates delamination during rifting. To illustrate this point, a similar simulation without an orogenic root is presented in Supplementary Figure 3. In that case, rifting does not trigger delamination, and no upwelling from the lower mantle is induced. Instead, mantle melting is predominantly driven by passive upwelling of the upper mantle, and melt production only reaches 4–5 km.

While numerous studies consider lower mantle upwelling as the cause for rifting and continental breakup, the results presented here suggest the reverse relationship, in which rifting and continental breakup trigger lower mantle upwelling. This means that plume activity does not need to be complementary to plate tectonic processes to explain anomalous magmatism. If a certain lower mantle region is not cooled by descending slabs and has not been tapped by mantle upwelling for sufficiently long time, a regional thermal anomaly that is preserved/protected by the convection-suppressing nature of the LUMB can develop. A regional mantle overturn can then be triggered by plate tectonic processes like the above-described mechanism. Our generalised model can explain the large rates of melt production associated with the formation of the NAIP and why excess magmatism still occurs in present-day Iceland. An improved model would require additional constraints on structure of the mantle beneath the North Atlantic region (e.g. ref.^37^), should be tested against geophysical and geochemical observations and would possibly also need to account for 3D effects involved in the process of delamination-induced mantle upwelling. Several other LIPs than the NAIP formed concurrently with continental breakup at locations of earlier suturing^10^. The model presented here provides a potential cause for this apparently common correlation. The implication would then be that mantle upwelling is not necessarily a process that operates outside of the context of plate tectonics, but is instead intimately related to the latter. Future work will address this question.

## Methods

### Overview

The employed numerical method for modelling visco-elastic-plastic flow is generally similar to that described by Gerya and Yuen^38^, but differs by employing a multigrid-based approach^24,39,40^ which allows for high resolution simulation in both space and time. The presented method also differs from the abovementioned references in that we employ a quantitative petrological model for the calculation of density and latent heat effects^41,42^.

The simulations of the present paper have a grid resolution of ~2 km for the entire 2000 km × 2000 km modelling domain and are run for 400 Myr.

The method employs a combined particle-in-cell approach^38^ for the coupled equations for the conservation of energy, momentum and mass in 2 dimensions, *x*_*i*_ (i = 1, 2):

1 $$\frac{\delta }{\delta {x}_{j}}(-k\frac{\delta T}{\delta {x}_{j}})=-\,\rho {C}_{P}\frac{DT}{Dt}+{H}_{r}+{H}_{a}+{H}_{S}+{H}_{L}-\rho L\frac{DF}{Dt}$$

2 $$\frac{\delta \sigma {^{\prime} }_{ij}}{\delta {x}_{j}}+\rho {g}_{i}-\frac{\delta P}{\delta x}=0$$

3 $$\frac{\partial {v}_{j}}{\partial {x}_{j}}=0$$

*k* is thermal conductivity, *T* is temperature, *C*_*P*_ is isobaric heat capacity, *ρ* is density and $\frac{DT}{Dt}$ is material time derivative of temperature. The source terms, *H*_*r*_ + *H*_*a*_ + *H*_*S*_ + *H*_*L*_ represent the total rate of enthalpy change due to radiogenic, adiabatic, shear, and solid state phase change heating, respectively. $\frac{DF}{Dt}$ is the material time derivative of the total melt depletion of the mantle source, and *L* is enthalpy change due to melting. $\sigma {^{\prime} }_{ij}$ is the deviatoric stress tensor defined as $\sigma {^{\prime} }_{ij}={\sigma }_{ij}+{\delta }_{ij}P$, where *σ*_*ij*_ is the stress tensor, and $P=-\frac{1}{2}{\sigma }_{kk}-\frac{1}{2}({\sigma }_{11}+{\sigma }_{22})$ is pressure. *v*_*j*_ is the velocity field vector that is constitutively related to stress through a viscous-elastic-plastic strain rate tensor (see the section on the rheological model in the below).

### Petrologic and thermodynamic model

To account for solid state phase changes at given pressure and temperature conditions, phase equilibria are calculated using the Gibb’s free energy minimisation software, Perple_X^42^ together with a thermodynamic database^36^ for common mantle mineral assemblages. We employ 3 general lithologies with different compositions in the Na_2_O-CaO-FeO-MgO-Al_2_O_3_-SiO_2_ (NCFMAS) system. These include (1) pyrolite^43^ for the ambient upper and lower mantle (with the exception of the mantle lithosphere and pyrolite that is subject to melt depletion during model evolution – see below equations 9 and 10, (2) mid-ocean ridge basalt, MORB^44,45^ for orogenic lower crust and mafic material in the transition zone^25^ and (3) Continental crust^44,46^. Compositions are given in Supplementary Table S2.

All endmembers and solution models are employed from the Stixrude and Lithgow-Bertelloni-database^36^ are employed, assuming the ‘Gt’ solution model for garnet.

To reduce computation time, the phase-equilibria are pre-calculated in Perple_X, and lookup tables for density (*ρ*), isobaric heat capacity (*C*_*P*_), and entropy of formation (*S*) are calculated on a grid in *P-T*-space with a resolution of 75 *MPa* and 10 *K*, respectively. In our numerical model, the tables are pre-loaded, and bilinear interpolation in *P-T*-space allows for efficient look-up of the relevant physical properties. To account for solid state phase changes and melting, we employ a method that can be regarded as an extension of any given thermo-mechanical method that otherwise does not account for such phase changes.

Density and isobaric heat capacity are calculated at each Lagrangian particle that has one of the three abovementioned lithologies and pressure, *P*_0_, and temperature, *T*_0_. Each time step is broken into two sub-steps where effects of solid state phase changes are accounted for. A third step accounts for effects of mantle melting.

#### Isentropic advection

The coupled momentum and continuity equations (2 and 3) are solved, and particles are then moved according to the calculated velocity field. This step involves a pressure change at each marker: *dP* = *P*_1_ − *P*_0_, where *P*_1_ is the new pressure. The associated temperature change due to adiabatic heating is found by assuming that entropy is conserved during the pressure change: *S*(*P*_0_, *T*_0_) = *S*(*P*_1_, *T*_1_). This equation is solved for *T*_1_ by Newton-Raphson iterations that are evaluated by calculating *S*(*P*_1_, *T*_1_) from repeated table interpolations with varying temperature. If Newton-Raphson iterations do not converge (e.g. in the case of steep entropy gradients with respect to temperature), a solution is found by using bisection starting at an interval that corresponds to the entire temperature range of the entropy table.

#### Isobaric thermal diffusion

This step involves the solution of the energy equation (1) using a procedure that has been employed in earlier works^24,39^, but differs by assuming that initial temperature is *T*_1_.

First, the energy equation (1) is solved for a time step *dt*, by assuming an initial temperature field *T*_1_ and that source terms for adiabatic and latent heating (due to both melting and solid state phase changes) are 0. The resulting time integrated temperature, *T*_2_, accounts for thermal diffusion, radiogenic heating and shear heating. The updated temperature *T*_2_ also implicitly accounts for adiabatic heating as this was calculated in the above step (1). To also account for latent heat effects due to solid state phase changes, a correction is applied by calculating the entropy change *dS* corresponding to the temperature difference between *T*_2_ and *T*_1_: $dS=\frac{dQ}{T}\approx {C}_{P}\frac{{T}_{2}-{T}_{1}}{{T}_{1}}$

This entropy change does not necessarily correspond to a change of sensible heat, only, but could also be associated with latent heating. Therefore the entropy table is employed to solve *S*(*P*_1_, *T*_1_) + *dS* = *S*(*P*_1_, *T*_3_) for *T*_3_. Like in the advection step, Newton-Raphson iterations or bisection are employed together with repeated entropy table interpolations.

#### Isobaric mantle melting

The thermodynamic database employed for the calculations in the above does not include a melt phase. Instead melt productivity is calculated following a procedure described elsewhere^39,47^. It is summarized here:

We employ a parameterization of mantle melting, where, the temperature *T*_*m*_ of a mantle peridotite source in equilibrium with melt is given as a function of pressure *P*_1_ and degree of melt depletion *F*. Here, we specifically employ the *T*_*m*_ -parameterisation for batch melting given by Katz *et al*.^48^, and assume a water content of 0 %, and an initial modal clinopyroxene concentration of 17 % for unmolten solid mantle. The change in the degree of melting is calculated by assuming an isobaric process where an excess temperature, *ΔT* = *T*_3_ − *T*_*m*_, due to the above steps 1-2, provides an entropy excess $d{S}_{steps1,2}=\frac{{{\rm{C}}}_{{\rm{P}}}{\rm{\Delta }}T}{{T}_{3}}$ that is assumed to balance the combined entropy increase due to both changing the degree of melting and temperature, respectively, $\frac{{{\rm{C}}}_{{\rm{P}}}{\rm{\Delta }}T}{{T}_{3}}=\frac{\partial S}{\partial F}{\rm{d}}F+\frac{\partial S}{\partial T}{\rm{d}}T$. This equation can be approximately solved^39,49^ for the increase of the degree of melt depletion, *ΔF*, on each marker as:

4 $${\rm{\Delta }}F=\frac{{\rm{\Delta }}T}{\frac{L}{{C}_{P}}+\frac{\delta Tm}{\delta F}}$$

Latent heating is given by *L* = *T*_3_Δ*S*_*melt*_, where Δ*S*_*melt*_, the entropy change due to melting, is assumed to be constant and independent of the degree of melting^49^. Melting is assumed to be irreversible at time scales greater than individual time steps, and the degree of melting therefore does not decrease if Δ*T* < 0. This assumption is based on yet another simplifying assumption that melts are rapidly removed from the source^50^, even though the melting parameterization is based on batch melting experiments. The calculated melt productivity can therefore only be regarded as a rough approximation.

Following Phipps Morgan & Morgan^49^, the temperature change due to latent heat consumption during melting is updated in accord with the change of *T*_*m*_ due to the melt increment *ΔF*, and the final temperature corrected for both melting and solid state phase changes is therefore (if *ΔF* > 0):

5 $${T}_{4}={T}_{m}+\frac{\delta {T}_{m}}{\delta F}\Delta F$$

If *ΔF* > 0, the final temperature is simply *T*_3_.

### Rheological model

Strain rate is related to the velocity field by $\dot{\in }{^{\prime} }_{ij}=\frac{1}{2}(\frac{\delta {v}_{i}}{\delta {x}_{j}}+\frac{\delta {v}_{j}}{\delta {x}_{i}})$ and is assumed to be a mutual contribution from brittle, elastic and viscous deformation, where both diffusion and dislocation creep^24,38^ are permitted:

6 $$\begin{array}{ccc}\dot{\in }{^{\prime} }_{ij} & = & \dot{\in }{^{\prime} }_{ij(diffusion)}+\dot{\in }{^{\prime} }_{ij(dislocation)}+\dot{\in }{^{\prime} }_{ij(elastic)}+\dot{\in }{^{\prime} }_{ij(plastic)}\\  & = & \frac{1}{2{\eta }_{(diffusion)}}\sigma {^{\prime} }_{ij}+\frac{1}{2{\eta }_{(dislocation)}}\sigma {^{\prime} }_{ij}+\frac{1}{2\mu }\frac{D\sigma {^{\prime} }_{ij}}{Dt}+\chi \frac{\sigma {^{\prime} }_{ij}}{{\sigma }_{II}}\end{array}$$

The plastic multiplier, *χ* is nonzero only if the second invariant of the deviatoric stress tensor, ${\sigma }_{II}=\sqrt{{\sigma }_{kk}}$, reaches the pressure-dependent Mohr-Coulomb failure limit:

7 $${\sigma }_{II}=C+P\,{\sin }(\varphi )$$

Where *C* is cohesion.

Viscosity depends on composition, pressure and temperature:

8 $$\eta =\frac{d(F)}{2A{\sigma }_{II}^{n-1}}{\exp }(\frac{E+PV}{RT})$$

*d*(*F*) is a depletion-dependent strengthening factor (see below).

For the two non-pyrolite lithologies, a simple, power-law rheology is however assumed for the viscous strain rate component with *d*(*F*) = 1 and $\dot{\in }{^{\prime} }_{ij(diffusion)}=0$. The continental crust follows a ‘plagioclase’ power-law^51^. The mafic lower crust initially present in the orogen at the centre of the model and within the mantle transition zone is assumed to follow an eclogite power-law^52^ at pressures less than 17.5 *GPa*. At higher pressures, a garnetite^53^ flow law is assumed.

For the pyrolite upper mantle, flow laws^54^ for both disclocation and diffusion creep are employed. For the pyrolite lower mantle, a simple linear flow law constrained by slab sinking rates^55^ is employed (assuming that $\dot{\in }{^{\prime} }_{ij(diflocation)}=0$). A density threshold of $4300\,\frac{kg}{{m}^{3}}$ is used to discriminate between upper and lower mantle rheology for pyrolite. The procedure for calculating density is described in the above section. Mechanical parameters are given in Table S1.

The presence of interstitial melt reduces the effective viscosity of mantle rocks^56^. However, the change the composition of the mantle source upon melt extraction tends to increase viscosity^56,57^. This strengthening effect is assumed to be of a larger magnitude than that of melt weakening, as we assume that melt is continuously extracted from the mantle source^47,58^. Following Phipps Morgan^57^, we assume two components of strengthening due to melt depletion (at a given pressure and temperature): (1) A 30-fold viscosity increase during the first 5% of melting where most water extraction is expected to occur, and (2) A third of an order of magnitude increase for each 15% increment of melt extraction due to the generally higher viscosity of refractory mantle with higher Mg-content. These effects are approximated by assuming that the depletion strengthening factor, *d*(*F*), is:

9 $$d(F)=(1+29\,{\rm{erf}}\,(\mathrm{20}\,F))\,\exp \,(8{\rm{.02}}\,F)$$

This is consistent with the parameterisation of Phipps Morgan^57^, and approximately consistent with the viscosity-depletion increase employed by Afonso *et al*.^59^ even though the latter only assume the effect of dehydration of viscosity.

Melt depletion is also known to decrease the density of the mantle residue^57,60^. This effect is approximated by correcting the density, *ρ*_(*Gibbs*)_, calculated from Gibb’s free energy minimisation:

10 $${\rho }_{(corrected)}={\rho }_{(Gibbs)}\,(1-0.0591\,F)$$

Following the Boussinesq approximation^51^, density changes due to changing pressure and temperature conditions are assumed to affect only the momentum equation (2), but are ignored in the continuity equation (3). Similarly, mass exchange between solid and melt phase and the relative advection between the two phases (compaction) is not accounted for in the continuity equation^47^.

### Thermal conductivity and radiogenic heat productivity

For continental crustal material, constant values of thermal conductivity are assumed (see Table S1). For pyrolite and for the MORB material assumed in the orogenic lower crust and in the mantle transition zone, a pressure and temperature-dependent parameterisation^61^ is assumed:

11 $$k={k}_{298}{(\frac{298k}{T})}^{q}\,(1+aP)$$

Here *q* is an exponent assumed to be 0.406 for both pyrolite and MORB. Similarly, for both compositions, a constant pressure gradient, $a=0.04\,GP{a}^{-1}$ is assumed^62^. The standard conductivity value, *k*_298_, is however assumed to differ between pyrolite and MORB compositions, such that for pyrolite at all conditions and MORB at pressures less than 17.GPa, it is ${k}_{298}=4.08\frac{J}{m\cdot K}$. At higher pressure, MORB is assumed behave similarly to a garnet rich assemblage that has a conductivity^63^ of ${k}_{298}=2.3\frac{J}{m\cdot K}$.

Radiogenic heat production is assumed to occur in the crust at a rate of ${H}_{r}=1.0\cdot {10}^{-6}\frac{W}{{m}^{3}}$. For MORB material, heat production is assumed to be negligible due to both small amounts and low productivities. Due to the great volumes of pyrolite, a nonzero heat production of ${H}_{r}=1.0\cdot {10}^{-12}\frac{W}{kg}\cdot \rho$ is assumed. This heat production corresponds to that reported for an average MORB source in Table 9 of ref.^32^ and references therein.

### Initial model state and boundary conditions

The model domain is a 2000 km by 2000 km square box (Fig. S1). A free surface is approximated using a weak ‘sticky air’ layer^64^ in the upper 80 km of the domain. The sticky air is visco-plastic with a viscosity of 10^20^ *Pa s* and a finite strength of 0.1 *MPa* (similar to the numerical precision of the modelling method). This renders the surface effectively stress-free even during rapid surface movements and dynamically limits the effective viscosity only when needed^39^. Below the sticky air is a layer of continental crust with a thickness of 40–50 km. The crustal thickness is 50 km in a 600 km wide region in the lateral centre of the model domain. Elsewhere, the crust is 40 km. Below, the thickened crust is a 20 km layer of MORB-like material. For reference, a model without an initial lower crustal MORB layer is calculated (Extended data Fig. 3). The rest of the model domain consists of mantle with a pyrolite composition with two exceptions:

1. A lithosphere/asthenosphere boundary is assumed at a depth of 150 km. Mantle within the lithosphere is possibly more depleted, stronger and more buoyant than the asthenospheric mantle below^57,59,60^. To approximate this, it is assumed that lithospheric mantle is depleted by *F* = 30%. This has the effect that the mantle lithosphere has ~1.8% less density than pyrolite at similar conditions (equation 10), and ~330 times higher viscosity (equation 9). Consistently with previously published models of long-term stability of continental lithosphere^65–67^, the mantle lithosphere remains stable against small-scale convective erosion during the first 300 Myr years of model evolution prior to rifting and retains an approximately constant thermal structure (Fig. 1).
2. At the mantle transition zone, a layer of MORB-material^25^ is imposed. The thermodynamic model implies that this material is neutrally buoyant relative to pyrolite at this depth^26^. This is because MORB-compositions are garnet rich and therefore denser than pyrolite above the 660 km discontinuity (where pyrolite is predominantly rich in ringwoodite), but less dense below the discontinuity where pyrolite is a perovskite-rich assemblage. Further below (ca. 100 km, depending on temperature), the MORB composition is again denser than pyrolite, due to garnet to perovskite transformation. Rather than imposing the garnetite layer at a fixed depth, we allow for it to form at a level of neutral buoyancy by initializing this material as a number of circular bodies that are capable of freely moving due to their buoyancy. They are initially placed at a depth of 660 km with 200 km lateral distance between their centres and have a radius of 50 km (Fig. S1). Within the first Myr of model evolution, they sink to a level of neutral buoyancy and spread out to a single layer due their finite viscosity.

#### Initial thermal structure and thermal boundary conditions

The temperature structure of the sub-lithospheric mantle is assumed to be initially adiabatic and is calculated as the contour (isentrope) of the pyrolite entropy map that intersects the surface pressure (*P* = 1 *Pa*), at a temperature of 1325 °C. At depths greater than 660 km, temperatures are elevated relative to this adiabat by 200 °C (Fig. S1), assuming that mantle convection is regionally in a layered state^21,22^ with a thermal boundary layer separating the upper and lower mantle (see also discussion in main paper). The 150 km lithosphere is assumed to be in a 2D conductive thermal steady state with the adiabatic mantle temperature as a lower boundary condition and 0 °C as an upper boundary. Vertical boundaries at the side of the model are everywhere isolating with zero lateral heat flow. The lower boundary is a constant temperature condition that equals the initial temperature at that depth.

#### Mechanical boundary conditions

During the first 300 Myr model time, the model has free slip at all 4 bounding sides and closed sides. This means that velocity perpendicular to a side is 0, and that traction parallel to the side is also zero. At 300 Myr, rifting of the lithosphere is kinematically forced by imposing plate separation, *v*_*sep*_, at a rate of *v*_*sep*_ = 1 *cm*/*yr*. This is employed by imposing outwards perpendicular velocities at both left and right boundaries of $\frac{1}{2}{v}_{sep}$ at depths from 0 to 240 km. To obey the volume-conserving continuity equation (3) globally, an inwards perpendicular flow of 0.4 cm/yr is imposed at the same boundaries at depths from 240 to 540 km. The material flowing into the model domain during the rift stage is pyrolite with *F* = 0% and a temperature that corresponds to the initial adiabat.

### Modelled melt productivity

The intensity of melt generation is calculated following an approach^68^ where an effective igneous crustal thickness is found by calculating the total mass of melt (per meter perpendicular to the 2D model cross section) produced during a given time step by integrating the change of depletion, Δ*F*, in a given time step, Δ*t*, over the whole modelling domain: ${M}_{melt}=\iint {\rm{\Delta }}F{\rho }_{source}dxdz\approx {\rho }_{source}\iint {\rm{\Delta }}Fdxdz$, where ${\rho }_{source}$ is an average density of the melt source region. If this mass of melt would be extracted to form igneous crust with average density, ${\rho }_{crust}$, its volume (i.e. area because the model is 2D) would be

12 $${V}_{crust}=\frac{{M}_{melt}}{{\rho }_{crust}}=\frac{{\rho }_{source}}{{\rho }_{crust}}\iint {\rm{\Delta }}F\,dxdz$$

If the total amount of plate separation during a time step, would be accommodated by the formation of a single dike with a width of *v*_*sep*_∆*t*, and if the total volume of crust produced would be emplaced within that dike as a single column, the effective igneous crustal thickness, *h*_*c*_, can be defined as the corresponding height of that column:

13 $${h}_{c}=\frac{1}{{v}_{sep}{\rm{\Delta }}t}\frac{{\rho }_{source}}{{\rho }_{crust}}\iint {\rm{\Delta }}F\,dxdz$$

This measure of productivity corresponds to the left y-axis of Fig. 3. Observationally constrained North Atlantic productivity estimates used in this study are not directly reported as magmatic crustal thickness values (e.g. in meters), but instead reflect the cross-sectional area of melt generated within certain (rift-perpendicular) profiles pr. time and are reported as e.g. km^3^/km/Ma^1^. To compare such data with modelling results, modelled productivities are also calculated as

14 $$\frac{d{V}_{crust}}{dt}=\frac{1}{{\rm{\Delta }}t}\frac{{\rho }_{source}}{{\rho }_{crust}}\iint {\rm{\Delta }}F\,dxdz$$

For the modelled productivities in Fig. 3, this rate corresponds to the right y-axis, and happens to be proportional to magmatic crustal thickness (equation 13), because the imposed boundary plate separation velocity, *v*_*sep*_, does not change at times where melts are produced.

The ratio of melt source density and melt density is assumed to be $\frac{{\rho }_{source}}{{\rho }_{crust}}=\frac{3300\frac{kg}{{m}^{3}}}{3000\frac{kg}{{m}^{3}}}$.

The modelled melt productivity is shown in Fig. 3 where it is decomposed into melt originating from a source that was initially (at the model onset time) part of the upper or lower mantle, respectively. This is specifically archived by splitting the integral $\iint {\rm{\Delta }}F\,dxdz$ appearing in equations (13 and 14) into two terms that each either integrate over the domain comprised of upper mantle material or the domain comprised by lower mantle material, respectively.

### North Atlantic melt productivity curves in Fig. 3

The North Atlantic melt productivity curves shown in Fig. 3 are based on refs^1,2,4^. The two curves referring to Holbrook *et al*.^1^ are based on the conjugate transects SIGMA I-Faroe Iceland Ridge and SIGMA II shown in their Fig. 5. Productivity is reported in units of volume pr. length pr. time, and Fig. 3 shows these curves without any further conversion. The productivity curve from Storey *et al*.^2^ is similarly presented as is in the original paper. The curves referring to Mjelde & Faleide^4^ are based on their Table S2 where productivities for Greenland-Faroe, Aegir/Kolbeinsey and Reykjanes ridge segments are given in units of volume pr. time. In the same table, characteristic lengths of each of these segments are given as 300, 500 and 1100 km, respectively. For comparison with model predictions and the other productivity curves, we normalise the volumetric productivities by these lengths to present productivities given in terms of volume pr. length pr. time. Furthermore, Mjelde & Faleide^4^ subtracted a ‘normal’ oceanic melt productivity of 5.5 km from their volumetric productivity estimates by assuming the time-dependent full-spreading rates given in their Table S1. We have re-added this background production, as our modelled melt productivities are not only a result of excess mantle upwelling and delamination, but also encompass melt generated from passive upwelling due to plate separation.

### Plate reconstructions shown in Fig. 3

Reconstructions, continent-ocean boundaries and Mid ocean ridges are extracted and modified from GPlates^69^. Dotted COB in Davis Strait indicates unconstrained lithospheric/crustal affinity (oceanic/continental). Red shaded areas indicate locations of contemporary magmatism at the following time periods: 100–65 Ma: ref.^70^. 65–50 Ma: refs^71,72^. 0 Ma: ref.^73^. From the latter, all units with ages <3.3 Ma are depicted as 0 Ma in our map. Reconstructed locations (stippled where extrapolated) of the Caledonian front (dark grey), the Iapetus suture (dark red), the Central Fjord (CF)-Flannan structure (FL) and Mona Lisa (ML) dipping structures (black lines with triangles indicating dip directions), active spreading ridges (red dashed lines) and abandoned spreading ridges (light grey dashed lines) are all based on refs^11,12,74^ and references therein.

## Electronic supplementary material

Supplementary notes, discussion and figures

Movie S1

## Data Availability

Numerical modelling code is available upon request from the authors.

## References

[1] Holbrook WS (2001). Mantle thermal structure and active upwelling during continental breakup in the North Atlantic. Earth and Planetary Science Letters.

[2] Storey M, Duncan RA, Swisher CC (2007). Paleocene-Eocene thermal maximum and the opening of the northeast Atlantic. Science.

[3] Saunders, A. D., Fitton, J. G., Kerr, A. C., Norry, M. J. & Kent, R. W. In *Large Igneous Provinces: Continental, Oceanic, and Planetary Flood Volcanism* 45–93 (American Geophysical Union, 1997).

[4] Mjelde R, Faleide JI (2009). Variation of Icelandic and Hawaiian magmatism: evidence for co-pulsation of mantle plumes?. Marine Geophysical Researches.

[5] Brown EL, Lesher CE (2014). North Atlantic magmatism controlled by temperature, mantle composition and buoyancy. Nature Geoscience.

[6] Mundl A (2017). Tungsten-182 heterogeneity in modern ocean island basalts. Science.

[7] Morgan WJ (1971). Convection Plumes in the Lower Mantle. Nature.

[8] Hofmann A (1997). Mantle geochemistry: the message from oceanic volcanism. Nature.

[9] French SW, Romanowicz B (2015). Broad plumes rooted at the base of the Earth's mantle beneath major hotspots. Nature.

[10] Buiter SJH, Torsvik TH (2014). A review of Wilson Cycle plate margins: A role for mantle plumes in continental break-up along sutures?. Gondwana Research.

[11] Schiffer C (2015). A sub-crustal piercing point for North Atlantic reconstructions and tectonic implications. Geology.

[12] Schiffer, C. *et al*. The Jan Mayen microplate complex and the Wilson cycle. *Geological Society, London, Special Publications* **470** (2018).

[13] Foulger GR (2002). Plumes, or plate tectonic processes?. Astronomy & Geophysics.

[14] King SD, Anderson DL (1998). Edge-driven convection. Earth and Planetary Science Letters.

[15] Korenaga J (2004). Mantle mixing and continental breakup magmatism. Earth and Planetary Science Letters.

[16] Elkins-Tanton LT (2005). Continental magmatism caused by lithospheric delamination. Geological Society of America Special Papers.

[17] Meyer R, van Wijk J, Gernigon L (2007). The North Atlantic Igneous Province: A review of models for its formation. Geological Society of America Special Papers.

[18] Schiffer, C., Balling, N., Jacobsen, B. H., Stephenson, R. A. & Nielsen, S. B. Seismological evidence for a fossil subduction zone in the East Greenland Caledonides. *Geology* (2014).

[19] Meissner R, Mooney W (1998). Weakness of the lower continental crust: a condition for delamination, uplift, and escape. Tectonophysics.

[20] Richter FM, McKenzie DP (1981). On some consequences and possible causes of layered mantle convection. Journal of Geophysical Research.

[21] Machetel P, Weber P (1991). Intermittent layered convection in a model mantle with an endothermic phase change at 670 km. Nature.

[22] Christensen UR, Yuen DA (1985). Layered convection induced by phase transitions. Journal of Geophysical Research: Solid Earth.

[23] Gerya, T. V. *Introduction to Numerical Geodynamic Modelling*. (Cambridge University Press, 2010).

[24] Petersen KD, Nielsen SB, Clausen OR, Stephenson R, Gerya T (2010). Small-Scale Mantle Convection Produces Stratigraphic Sequences in Sedimentary Basins. Science.

[25] Karato S-i (1997). On the separation of crustal component from subducted oceanic lithosphere near the 660 km discontinuity. Physics of the Earth and Planetary Interiors.

[26] Irifune T, Ringwood AE (1993). Phase transformations in subducted oceanic crust and buoyancy relationships at depths of 600–800 km in the mantle. Earth and Planetary Science Letters.

[27] Buck RW (1986). Small-scale convection induced by passive rifting: the cause for uplift of rift shoulders. Earth and Planetary Science Letters.

[28] White RS, McKenzie D, O'Nions RK (1992). Oceanic crustal thickness from seismic measurements and rare earth element inversions. Journal of Geophysical Research: Solid Earth.

[29] Davies Geoffrey F. (2008). Episodic layering of the early mantle by the ‘basalt barrier’ mechanism. Earth and Planetary Science Letters.

[30] Schiffer C, Nielsen SB (2016). Implications for anomalous mantle pressure and dynamic topography from lithospheric stress patterns in the North Atlantic Realm. Journal of Geodynamics.

[31] White N, Lovell B (1997). Measuring the pulse of a plume with the sedimentary record. Nature.

[32] Jaupart, C. & Labrosse, S. In *Treatise on Geophysics* Vol. 7 (ed. G. Schubert) 223–270 (Elsevier, 2015).

[33] Burke K, Steinberger B, Torsvik TH, Smethurst MA (2008). Plume Generation Zones at the margins of Large Low Shear Velocity Provinces on the core–mantle boundary. Earth and Planetary Science Letters.

[34] Heron PJ, Lowman JP (2011). The effects of supercontinent size and thermal insulation on the formation of mantle plumes. Tectonophysics.

[35] Cottaar S, Deuss A (2016). Large_scale mantle discontinuity topography beneath Europe: Signature of akimotoite in subducting slabs. Journal of Geophysical Research: Solid Earth.

[36] Stixrude L, Lithgow-Bertelloni C (2011). Thermodynamics of mantle minerals - II. Phase equilibria. Geophysical Journal International.

[37] Rickers F, Fichtner A, Trampert J (2013). The Iceland–Jan Mayen plume system and its impact on mantle dynamics in the North Atlantic region: Evidence from full-waveform inversion. Earth and Planetary Science Letters.

[38] Gerya TV, Yuen DA (2007). Robust characteristics method for modelling multiphase viscoelasto-plastic thermo-mechanical problems. Physics of the Earth and Planetary Interiors.

[39] Petersen K, Armitage J, Nielsen S, Thybo H (2015). Mantle temperature as a control on the time scale of thermal evolution of extensional basins. Earth and Planetary Science Letters.

[40] Petersen KD, Schiffer C (2016). Wilson cycle passive margins: Control of orogenic inheritance on continental breakup. Gondwana Research.

[41] Gerya TV, Connolly JA, Yuen DA, Gorczyk W, Capel AM (2006). Seismic implications of mantle wedge plumes. Physics of the Earth and Planetary Interiors.

[42] Connolly J (2005). Computation of phase equilibria by linear programming: a tool for geodynamic modeling and its application to subduction zone decarbonation. Earth and Planetary Science Letters.

[43] Ringwood A. E. (1979). Origin of the Earth and Moon.

[44] Massonne H-J, Willner AP, Gerya T (2007). Densities of metapelitic rocks at high to ultrahigh pressure conditions: What are the geodynamic consequences?. Earth and Planetary Science Letters.

[45] Schilling J (1983). Petrologic and geochemical variations along the Mid-Atlantic Ridge from 29 degrees N to 73 degrees N. American Journal of Science.

[46] McLennan, S. M. In *Rock Physics & Phase Relations* (ed. T. J. Ahrens) 8–19 (American Geophysical Union, 1995).

[47] Nielsen TK, Hopper JR (2004). From rift to drift: Mantle melting during continental breakup. Geochemistry, Geophysics, Geosystems.

[48] Katz RF, Spiegelman M, Langmuir CH (2003). A new parameterization of hydrous mantle melting. Geochemistry, Geophysics, Geosystems.

[49] Phipps Morgan J, Morgan WJ (1999). Two-stage melting and the geochemical evolution of the mantle: A recipe for mantle plum-pudding. Earth and Planetary Science Letters.

[50] McKenzie D, Bickle MJ (1988). The Volume and Composition of Melt Generated by Extension of the Lithosphere. Journal of Petrology.

[51] Ranalli, G. *Rheology of the Earth*. 2 edn, 413 (Chapman & Hall, 1995).

[52] Zhang J, Green HW (2007). Experimental Investigation of Eclogite Rheology and Its Fabrics at High Temperature and Pressure. Journal of Metamorphic Geology.

[53] Mei S, Suzuki AM, Kohlstedt DL, Xu L (2010). Experimental investigation of the creep behavior of garnet at high temperatures and pressures. Journal of Earth Science.

[54] Karato SI, Wu P (1993). Rheology of the upper mantle: A synthesis. Science.

[55] Čížková H, van den Berg AP, Spakman W, Matyska C (2012). The viscosity of Earth’s lower mantle inferred from sinking speed of subducted lithosphere. Physics of the earth and Planetary Interiors.

[56] Hirth G, Kohlstedt DL (1995). Experimental constraints on the dynamics of the partially molten upper mantle: 2. Deformation in the dislocation creep regime. Journal of Geophysical Research: Solid Earth.

[57] Phipps Morgan J (1997). The generation of a compositional lithosphere by mid-ocean ridge melting and its effect on subsequent off-axis hotspot upwelling and melting. Earth and Planetary Science Letters.

[58] Ito G, Shen Y, Hirth G, Wolfe CJ (1999). Mantle flow, melting, and dehydration of the Iceland mantle plume. Earth and Planetary Science Letters.

[59] Afonso JC, Zlotnik S, Fernàndez M (2008). Effects of compositional and rheological stratifications on small-scale convection under the oceans: Implications for the thickness of oceanic lithosphere and seafloor flattening. Geophysical Research Letters.

[60] Jordan TH (1978). Composition and development of the continental tectosphere. Nature.

[61] Xu Y (2004). Thermal diffusivity and conductivity of olivine, wadsleyite and ringwoodite to 20 GPa and 1373 K. Physics of the Earth and Planetary Interiors.

[62] Chai M, Brown J, Slutsky L (1996). Thermal diffusivity of mantle minerals. Physics and Chemistry of Minerals.

[63] Giesting P, Hofmeister A, Wopenka B, Gwanmesia G, Jolliff B (2004). Thermal conductivity and thermodynamics of majoritic garnets: implications for the transition zone. Earth and Planetary Science Letters.

[64] Crameri F (2012). A comparison of numerical surface topography calculations in geodynamic modelling: An evaluation of the ‘sticky air’method. Geophysical Journal International.

[65] Hieronymus CF, Shomali ZH, Pedersen LB (2007). A dynamical model for generating sharp seismic velocity contrasts underneath continents: Application to the Sorgenfrei–Tornquist Zone. Earth and Planetary Science Letters.

[66] Doin MP, Fleitout L, Christensen U (1997). Mantle convection and stability of depleted and undepleted continental lithosphere. Journal of Geophysical Research B: Solid Earth.

[67] Lenardic, A., Moresi, L. N. & Mühlhaus, H. Longevity and stability of cratonic lithosphere: insights from numerical simulations of coupled mantle convection and continental tectonics. *Journal of Geophysical Research: Solid Earth* **108** (2003).

[68] Ito G, Lin J, Gable CW (1996). Dynamics of mantle flow and melting at a ridge-centered hotspot: Iceland and the Mid-Atlantic Ridge. Earth and Planetary Science Letters.

[69] Müller RD, Sdrolias M, Gaina C, Roest WR (2008). Age, spreading rates, and spreading asymmetry of the world's ocean crust. Geochemistry, Geophysics, Geosystems.

[70] Wilkinson CM, Ganerød M, Hendriks BW, Eide EA (2017). Compilation and appraisal of geochronological data from the North Atlantic Igneous Province (NAIP). Geological Society, London, Special Publications.

[71] Abdelmalak MM (2012). Stress fields acting during lithosphere breakup above a melting mantle: A case example in West Greenland. Tectonophysics.

[72] Jones MT (2016). Provenance of bentonite layers in the Palaeocene strata of the Central Basin, Svalbard: implications for magmatism and rifting events around the onset of the North Atlantic Igneous Province. Journal of Volcanology and Geothermal Research.

[73] Jóhannesson, H. & Sæmundsson, K. (Geological Survey of Iceland, 2009).

[74] Abramovitz T, Thybo H (2000). Seismic images of Caledonian, lithosphere-scale collision structures in the southeastern North Sea along Mona Lisa Profile 2. Tectonophysics.

