# Supplementary material for: LIP formation and protracted lower mantle upwelling induced by rifting and delamination
Source: Sci Rep. 2018 Nov 8;8:16578. doi: 10.1038/s41598-018-34194-0 (PMC6224380; doi:10.1038/s41598-018-34194-0)
Supplement: Supplementary file 1 — Supplementary notes, discussion and figures [file 41598_2018_34194_MOESM1_ESM.docx]

*Supplementary information for*

**LIP formation and protracted lower mantle upwelling induced by rifting and delamination**

**Authors:** Kenni Dinesen Petersen^1^*, Christian Schiffer^2^, Thorsten Nagel^1^

**Affiliations:**

^1^Department of Geoscience, Aarhus University, 8000 Aarhus, Denmark

^2^Department of Earth Sciences, Durham University, Durham DH1 3LE, UK.

*Correspondence to: [kenni@geo.au.dk](mailto:kenni@geo.au.dk)

**Note on radiogenic heating rate**

For a stagnant lower mantle, conductive heat loss can be neglected at time scales less than that of thermal diffusion, $\tau$ (e.g. for a length scale of 2000 km and thermal diffusivity of 1 mm^2^s^-1^, $\tau=\frac{\left( 2000km \right)^{2}}{1mm^{2}s^{-1}}\approx120 Gyr$), and the radioactive heat production can be assumed to exclusively change the thermal energy, if latent energy changes are neglected. For a small volume $V$ this implies that the increase of heat energy, $dQ$, during a small instance of time, $dt$, is given by ${dQ=dtVH}_{r}=\rho VC_{p}dT$, where $dT$ is temperature increase. This implies that the rate of temperature change is:

$\frac{dT}{dt}=\frac{H_{r}}{\rho C_{p}}$ ()

For the depleted MORB source-like radioactive element composition^31^ assumed for heat production in the present study, where $\frac{H_{r}}{\rho}=1.0\cdot{10}^{-12}\frac{W}{kg}$, and by assuming a constant, specific heat capacity of 1000 $\frac{J}{kg\cdot K}$, the radiogenic heating is $\frac{dT}{dt}\approx88\frac{K}{Gyr}$. Assuming a radioactive concentration corresponding to the Bulk Silicate Earth estimate of reference ^31^ with $\frac{H_{r}}{\rho}=5.1\cdot{10}^{-12}\frac{W}{kg}$, we get $\frac{dT}{dt}\approx160\frac{K}{Gyr}$.

**Sensitivity study of modelling parameters**

In order to test the robustness of the conclusions based on the model presented in the main paper (hereafter referred to as the reference model), we ran numerical simulations using perturbed values of selected modelling parameters while keeping everything else constant. These supplementary models show the effect of different initial thicknesses of the mafic lower crust (0 and 10 km, respectively; Figs. S3-4); the effect of different initial temperature differences between the upper and lower mantle (0 and 100°C, respectively; Figs.S5-6); the effect of no MORB graveyard at the LUMB (Fig. S7); and the effect of changing onset time of extension (50 and 350 Myr, respectively; Figs. S8-9).

*Thickness of mafic lower crust*

An initial mafic lower crustal thickness of 20 km is assumed in the reference model. Fig. S3 shows a model where such a mafic lower crustal root is not present. In this case, extension leads to rifting and thinning of the mantle lithosphere, but delamination does not occur, as the average density of the mantle lithosphere is lower than in the reference model (Fig. 2). Consequently, upwelling of the lower mantle is not initiated as in the reference model, and the resulting melt productivity evolution is simply governed by passive upwelling of upper mantle material, and only reaches 4-5 km after breakup (Fig. S3i).

However, with only 10 km initial mafic lower crust (Fig. S4), delamination is induced by rifting, and lower mantle upwelling develops. In this case, delamination occurs later than in the reference model (~22 Myr after the onset of extension), and only involves one side of the rift zone. Consequently, the detailed melt productivity evolution (Fig. S4i) is different from that of the reference model, but reaches much higher rates than in the above case where delamination and lower mantle upwelling does not occur (Fig. S3).

The implication of the models shown in Fig. S3-4 and the reference model is that the presence of mafic lower crust is a necessary condition for rift-induced delamination and lower mantle upwelling in the context of the models presented in this paper. An additional implication is that our models do not (erroneously) predict ubiquitous LIP-formation during continental breakup. If no dense root is present in a rift developing along an older suture (perhaps it was lost earlier or never formed), melting during and after breakup is associated with passive upwelling of upper mantle, forming ‘normal’ oceanic crust with a thickness of less than 10 km. This can occur without disturbing the thermal stratification below, and rifting can therefore also take place above a relatively hot lower mantle without LIP formation.

*Potential temperature of the lower mantle*

For the reference model, an initial stratified thermal structure with a lower mantle 200 °C hotter than a pyrolite adiabat with a potential temperature of 1325 °C is assumed. Fig. S5 shows a model where no initial thermal stratification is assumed, and potential temperature of the sublithospheric mantle is 1325 °C everywhere. In this case, extension causes rift-induced delamination and associated high melt productivity as in the reference model. The delaminated material similarly sinks rapidly to the LUMB, but only slowly penetrates into the lower mantle and does not induce upwelling of the latter. The reason for this difference is the lack of thermal buoyancy, and the ~5 times higher viscosity of the lower mantle due to the lower temperature.

In the case of a lower mantle with 100 °C higher temperature (Fig. S6), delamination causes some lower mantle upwelling and associated higher melt productivity, but the rate of upwelling is lower than in the reference model.

These models (Fig. S5-6 and Fig.1) show that a relatively hot or buoyant lower mantle is required for delamination-induced mantle upwelling in the context of model assumptions presented here.

*MORB graveyard at the LUMB*

The reference model assumes the initial presence of MORB material at the LUMB boundary (Fig. S1). A model with no such material is shown in Fig. S7. In such a model, the assumed thermal stratification of the mantle is unstable, and convective instability develops within the first few Myr of the simulation. This is in contrast with the rest of the models presented in this paper where the presence of MORB at the LUMB stabilizes thermal stratification for several 100 Myr. This implies that the results presented in the main paper rely on the existence of such a layer or some other mechanism that tends to promote thermal stratification of the mantle. Another possibility of a mechanism that would tend to hamper convection through the MTZ is the effect of the displaced density jump the Ringwoodite-out transition due to its negative Clapeyron slope^74^. In the present paper, we employ the database of Stixrude & Lithgow-Bertelloni^35^ which predicts a relatively high slope of ca. -1.1 MPa/K for a pyrolite composition. However, numerous experiments, first-principle calculations and fundamental seismic studies point to values lower than -2 MPa/K (Figure 9 of ref ^34^). With such a lower slope, convective flow of pyrolite between the upper and lower mantle would tend to be more limited than in the models presented here and therefore favour layered or ephemerally layered convection. Our future work will look into the role of this phase transition in the context of delamination-induced mantle upwelling.

*Onset time of extension*

In the reference model, extension of the lithosphere is applied after 300 Myr of tectonic ‘quiescence’ with a vigorously convecting upper mantle and a relatively stagnant lower mantle. This situation is discontinued at the onset of extension, when rifting leads to delamination that disturbs mantle stratification and induces upwelling of the lower mantle. In Figs. S8-9 models with extensional onset at 50 Myr and 350 Myr, respectively, are presented. The evolution of both models is very similar to that of the reference model in the sense that rift-induced delamination and lower mantle upwelling occur within 9-18 Myr after the onset of extension. This demonstrates that extension is the cause for lower mantle upwelling and e.g. rules out the possibility that a convective instability is about to onset at 300 Myr in the reference model, whether or not extension is applied.

**Supplementary figures**


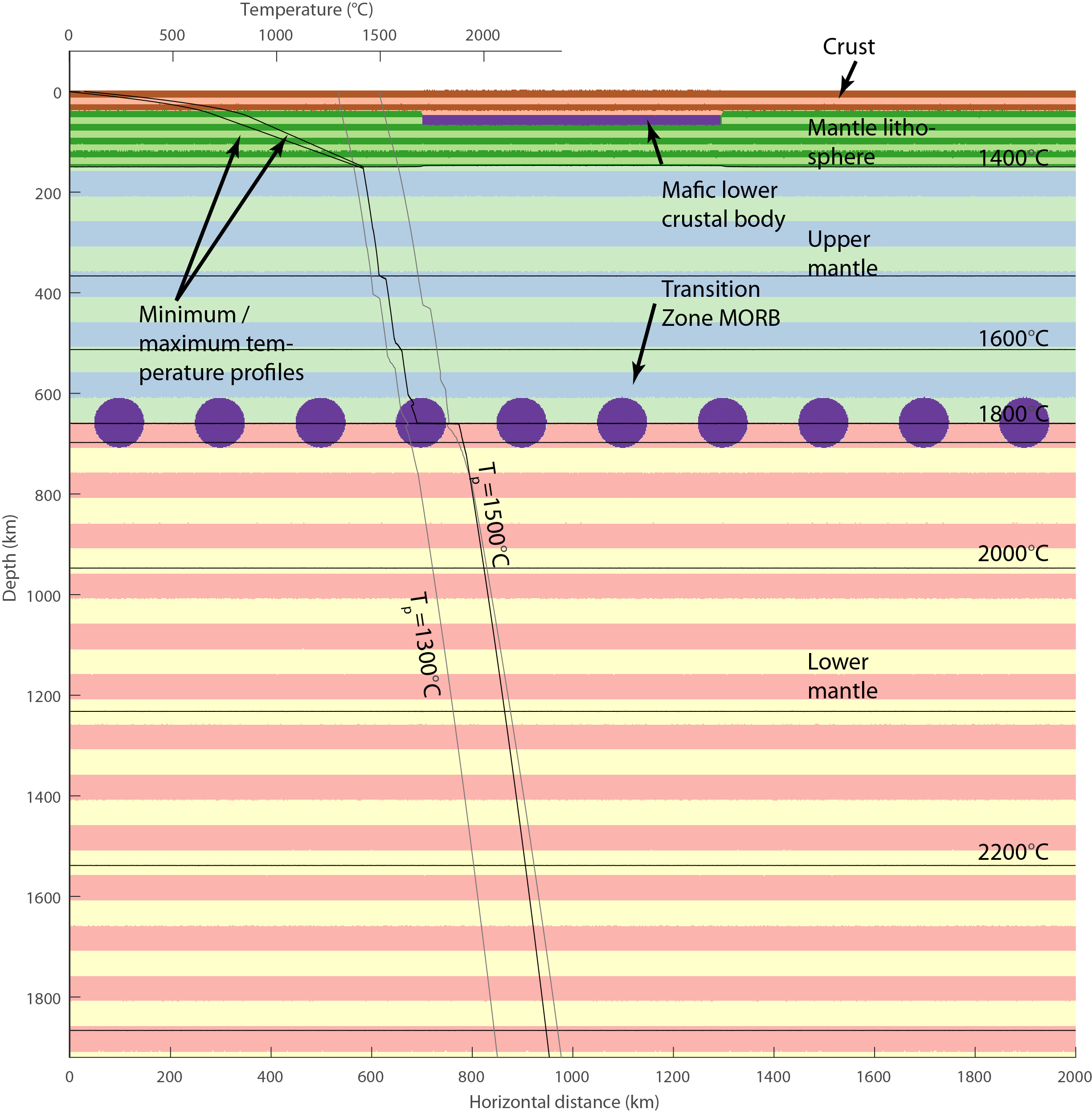


**Figure S1 | Initial state of model.** The initial position of assumed lithologies is indicated in different colors that are consistent with those shown in Fig. 1. Isotherms and temperature profiles are also consistent with Fig. 1. The initial minimum and maximum temperature profiles (bold black line) reflect the assumed initial thermal structure: For the upper mantle, an adiabat is assumed that corresponds to T_p_=1325 °C for a pyrolite composition and lithostatic pressure/density. At depths greater than 660 km, temperatures are assumed to be elevated relative to this adiabat by 200°C (Fig. S1). The thermal structure of the 150 km lithosphere is assumed to be in a 2D conductive thermal steady state with the adiabatic mantle temperature as a lower boundary condition and 0 °C as an upper boundary. The differences between maximum and minimum temperatures within the lithosphere are due to the thicker heat-producing crust in the centre of the modelling domain. The 10 circular bodies referred to as ‘Transition zone MORB’ have a diameter of 100 km and have centres at a depth of 660 km. Within the first Myr of the simulation, these MORB bodies sink to a level of neutral buoyancy and spread out to a single layer due their finite viscosity. This approach is preferred, because an initially horizontal layer could have non-neutral buoyancy and yet remain (quasi-)stable, because there would be no horizontal density gradients to drive flow. Furthermore and for similar reasons, the immediate gravitational reorganization of the MORB bodies also help initiate convection in the upper mantle that would otherwise be artificially stable due to the assumed 1D thermal structure.


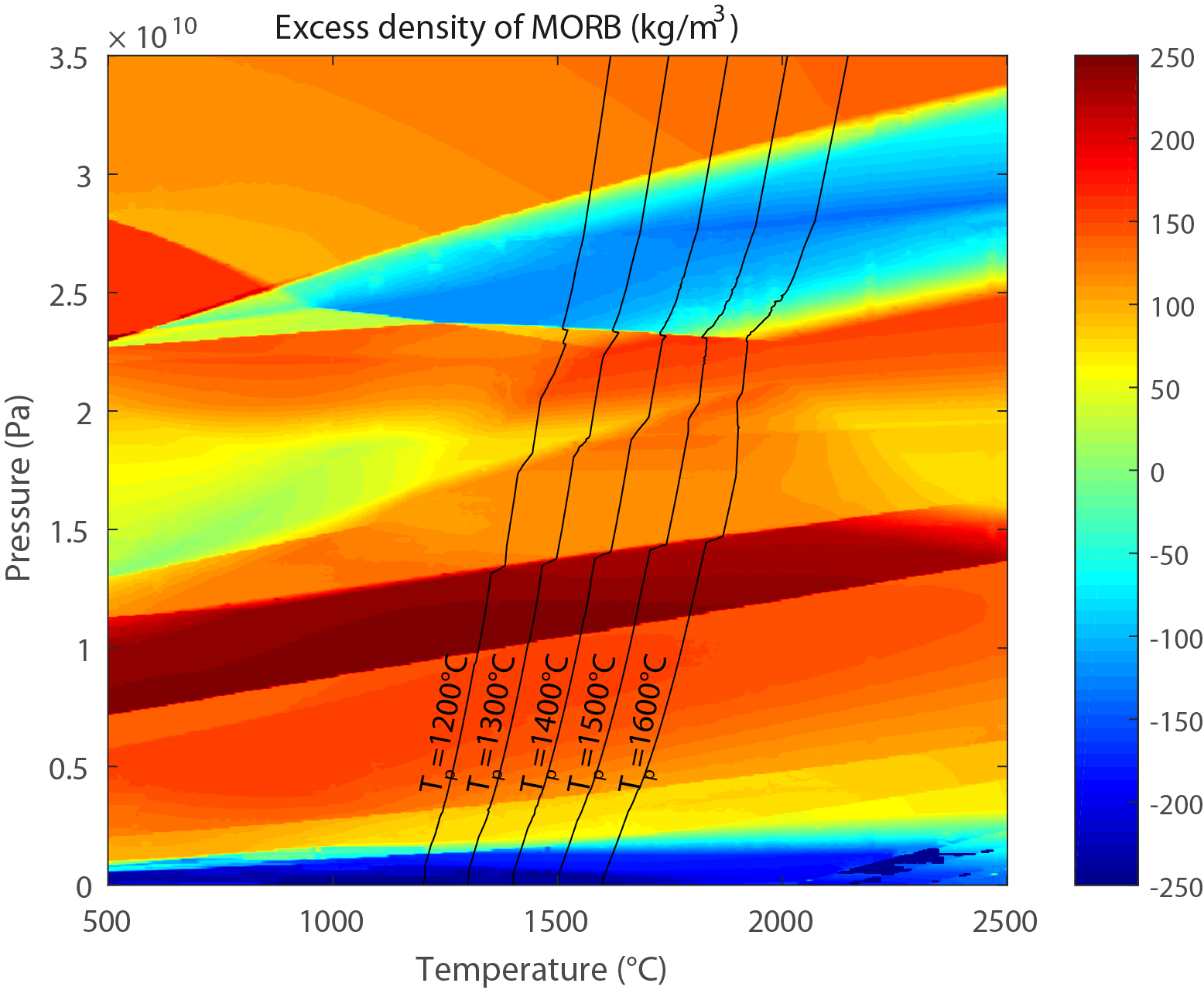


**Figure S2 | Temperature and pressure-dependent density differences between the MORB and pyrolite compositions employed in this study.** Black, superimposed curves show pyrolite adiabats (isentropes) for selected potential temperatures (T_p_). Density and entropy maps are calculated using Perple_X^41^ and the thermodynamic database of Stixrude and Lithgow-Bertelloni^35^.


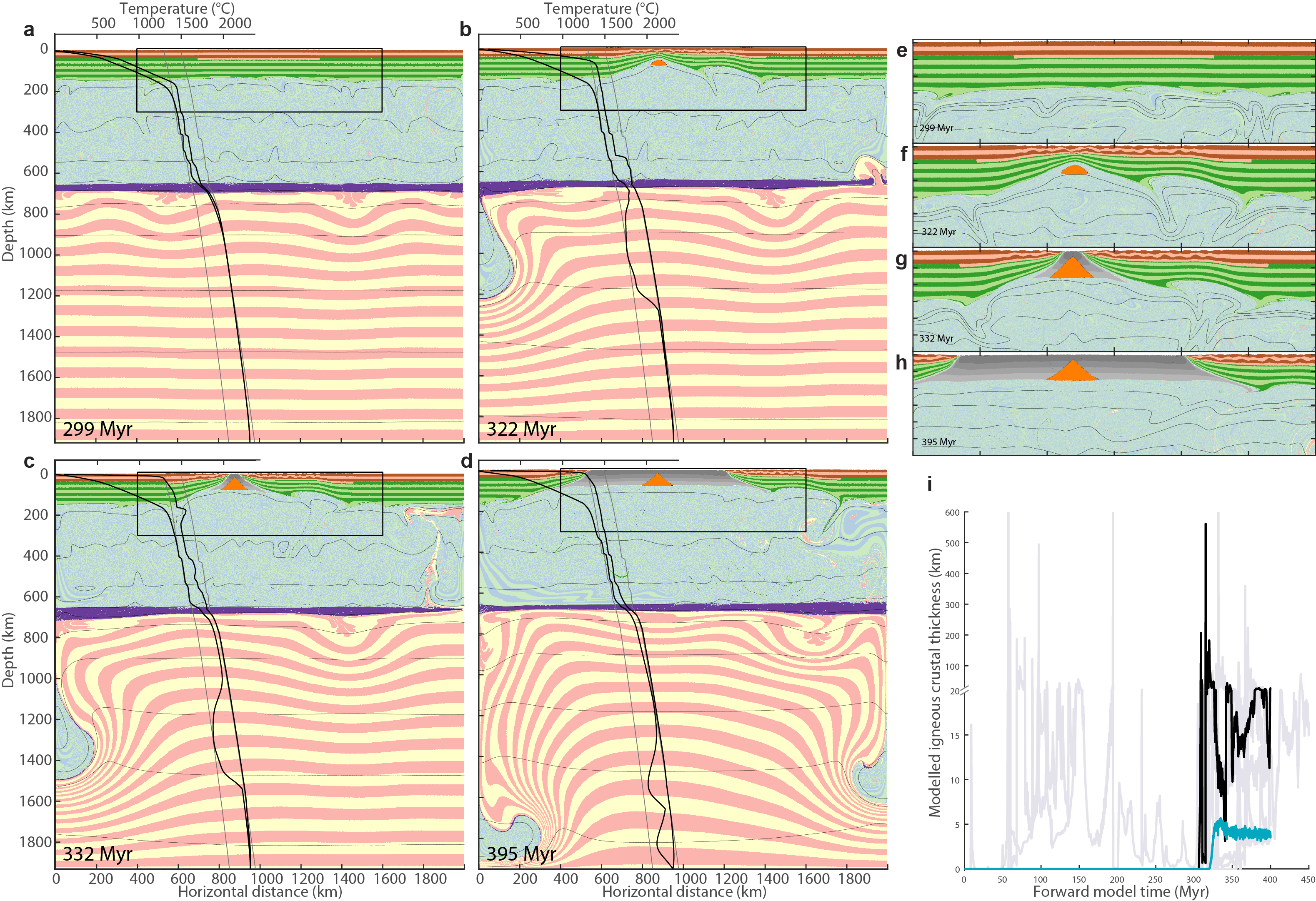


**Figure S3 | Model with no initial mafic lower crust.** The model is otherwise similar to the reference model. Colors and lines in (**a**-**d**) and the close ups in (**e**-**h**) indicate lithology, isotherms and temperature profiles in the same way as is in Fig. 1a-l. (**i**) Melt productivity of the corresponding model (a-h) is shown in blue and melt productivity of the reference model (e.g. Fig. 3) is shown in black for comparison. The melt productivity of all the other models presented in this supplement are shown in light grey. Note the broken y-axis at 20 km. The lack of of a mafic lower crust makes a substantial difference compared to the reference model, because rifting does not lead to delamination and associated mantle upwelling. Melt productivity is therefore much lower (up to 5 km) and governed by passive upwelling of upper mantle material with a potential temperature of 1300-1350 °C. During extension, material exchanges of small ‘blobs’ occur across the LUMB. These are caused by the kinematic boundary conditions imposed at the vertical sides of the model during extension (where an inwards flow is imposed at depths 240 to 540 km to preserve the volume lost by the imposed extension above). This blobs do not affect melt productivity.


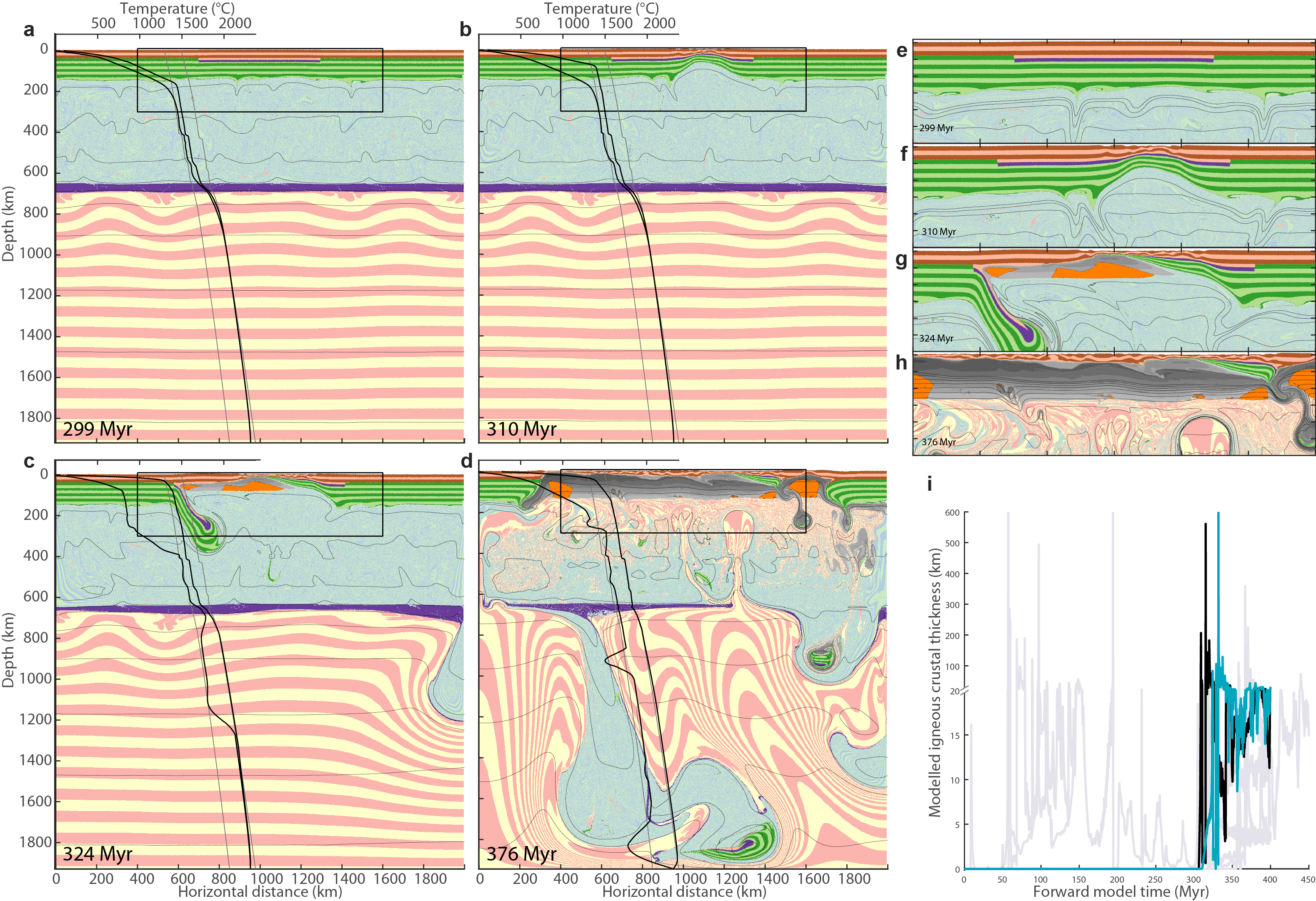


**Figure S4 | Model with 10 km initial mafic lower crust.** The model is otherwise similar to the reference model. Colors and lines in (**a**-**d**) and the close ups in (**e**-**h**) indicate lithology, isotherms and temperature profiles in the same way as is in Fig. 1a-l. (**i**) Melt productivity of the corresponding model (a-h) is shown in blue and melt productivity of the reference model (e.g. Fig. 3) is shown in black for comparison. The melt productivity of all the other models presented in this supplement are shown in light grey. Note the broken y-axis at 20 km. The presence of only 10 km mafic lower crust is sufficient for delamination during rifting and associated upwelling from the lower mantle. This model therefore generally evolves similarly to the reference model (that has an initial mafic lower crust of 20 km). A difference is that delamination here onset slightly later (at ~322 Myr) and only involves the lithosphere to the left of the rift (the right part partially delaminates much later at ~356 Myr). Consequently, the detailed melt productivity evolution (i) is different, but still reaches much higher rates than in the case where delamination and lower mantle upwelling does not occur (Fig. S3).


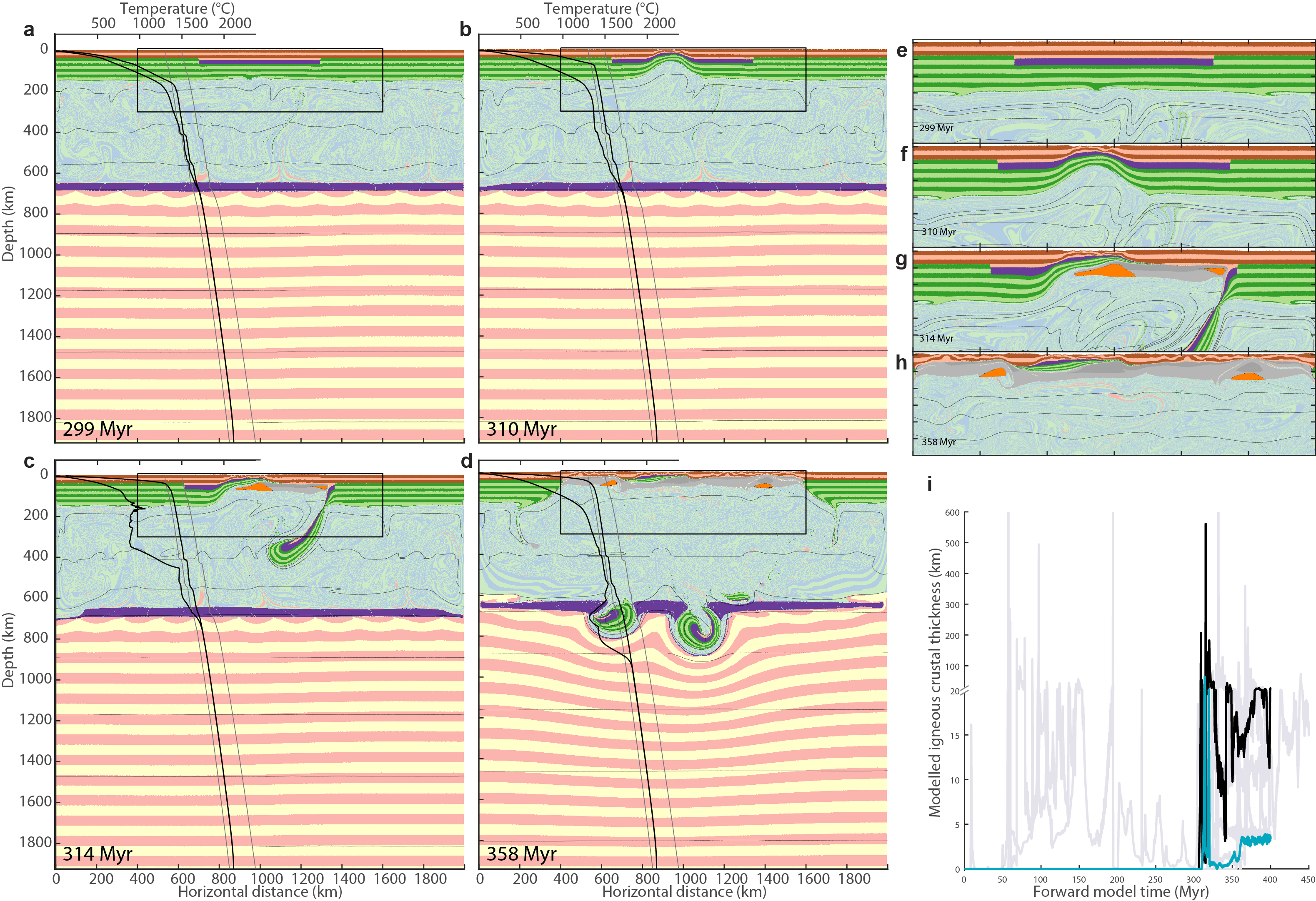


**Figure S5 | Model with potential temperature of the lower mantle identical to that of the upper mantle (1325 °C).** The model is otherwise similar to the reference model. Colors and lines in (**a**-**d**) and the close ups in (**e**-**h**) indicate lithology, isotherms and temperature profiles in the same way as is in Fig. 1a-l. (**i**) Melt productivity of the corresponding model (a-h) is shown in blue and melt productivity of the reference model (e.g. Fig. 3) is shown in black for comparison. The melt productivity of all the other models presented in this supplement are shown in light grey. Note the broken y-axis at 20 km. In this model, extension causes rift-induced delamination and associated high melt productivity as in the reference model. The delaminated material similarly sinks rapidly to the LUMB, but only slowly penetrates into the lower mantle and does not induce upwelling of the latter. The reason for this difference is the lack of thermal buoyancy, and ~5 times higher viscosity (compared to that of the reference model) of the lower mantle due to the lower temperature.

**Figure S6 | Model with potential temperature of the lower mantle 100 °C higher than that of the upper mantle.** The model is otherwise similar to the reference model. Colors and lines in (**a**-**d**) and the close ups in (**e**-**h**) indicate lithology, isotherms and temperature profiles in the same way as is in Fig. 1a-l. (**i**) Melt productivity of the corresponding model (a-h) is shown in blue and melt productivity of the reference model (e.g. Fig. 3) is shown in black for comparison. The melt productivity of all the other models presented in this supplement are shown in light grey. Note the broken y-axis at 20 km. Compared to the model presented in Fig. S5 with no temperature anomaly of the lower mantle, this model shows little delamination-induced lower mantle upwelling. However, compared to the reference model (with additionally 100 °C higher temperature of the lower mantle), where lower mantle upwelling results in almost complete replacement of the sublithospheric uppermost mantle and an associated high rate of melting, this colder model shows the arrival of individual lower mantle ‘blobs’ that results in individual peaks of melt productivity of 10-20 km. The average post-delamination productivity is ~6 times lower than that of the reference model.


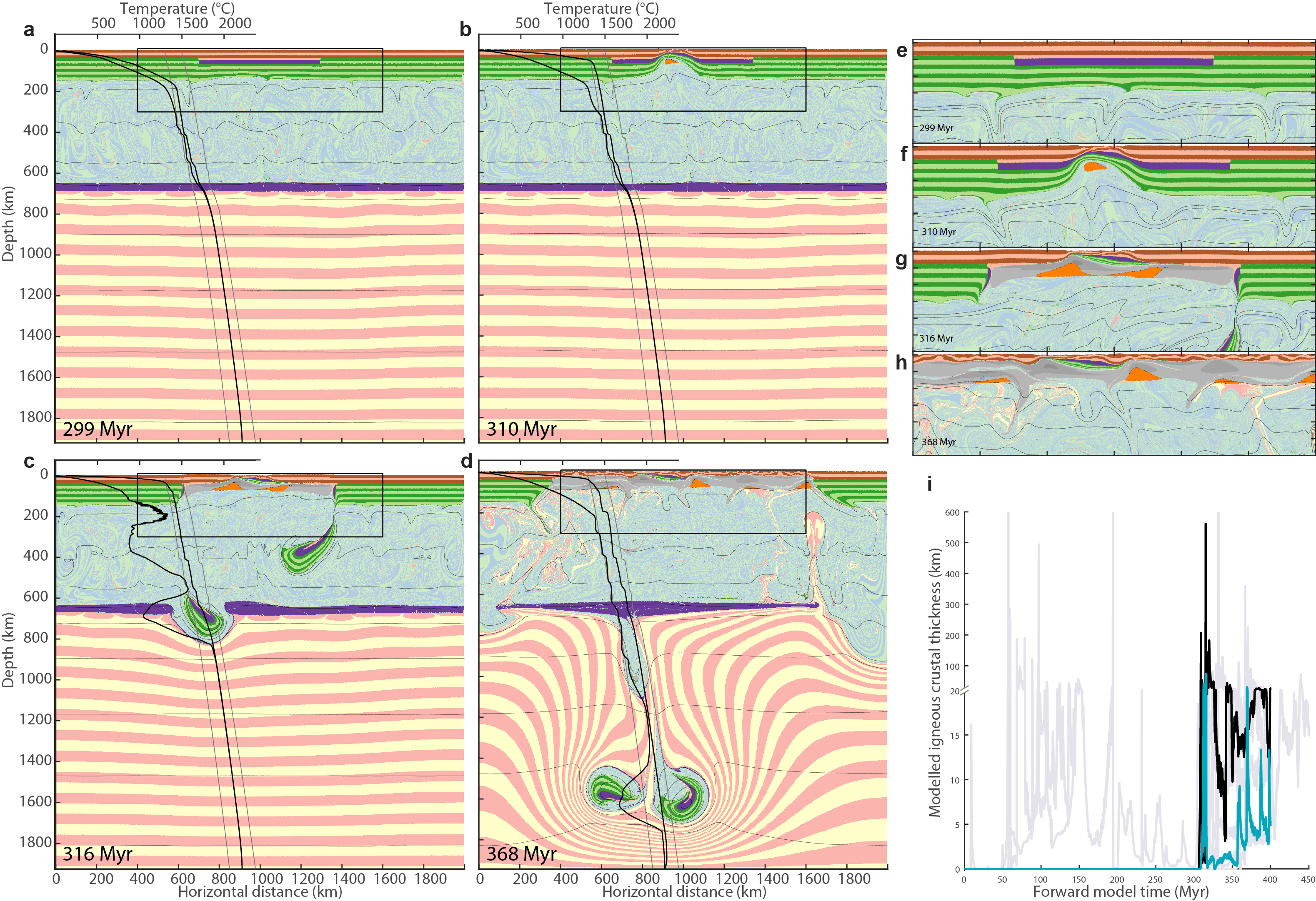

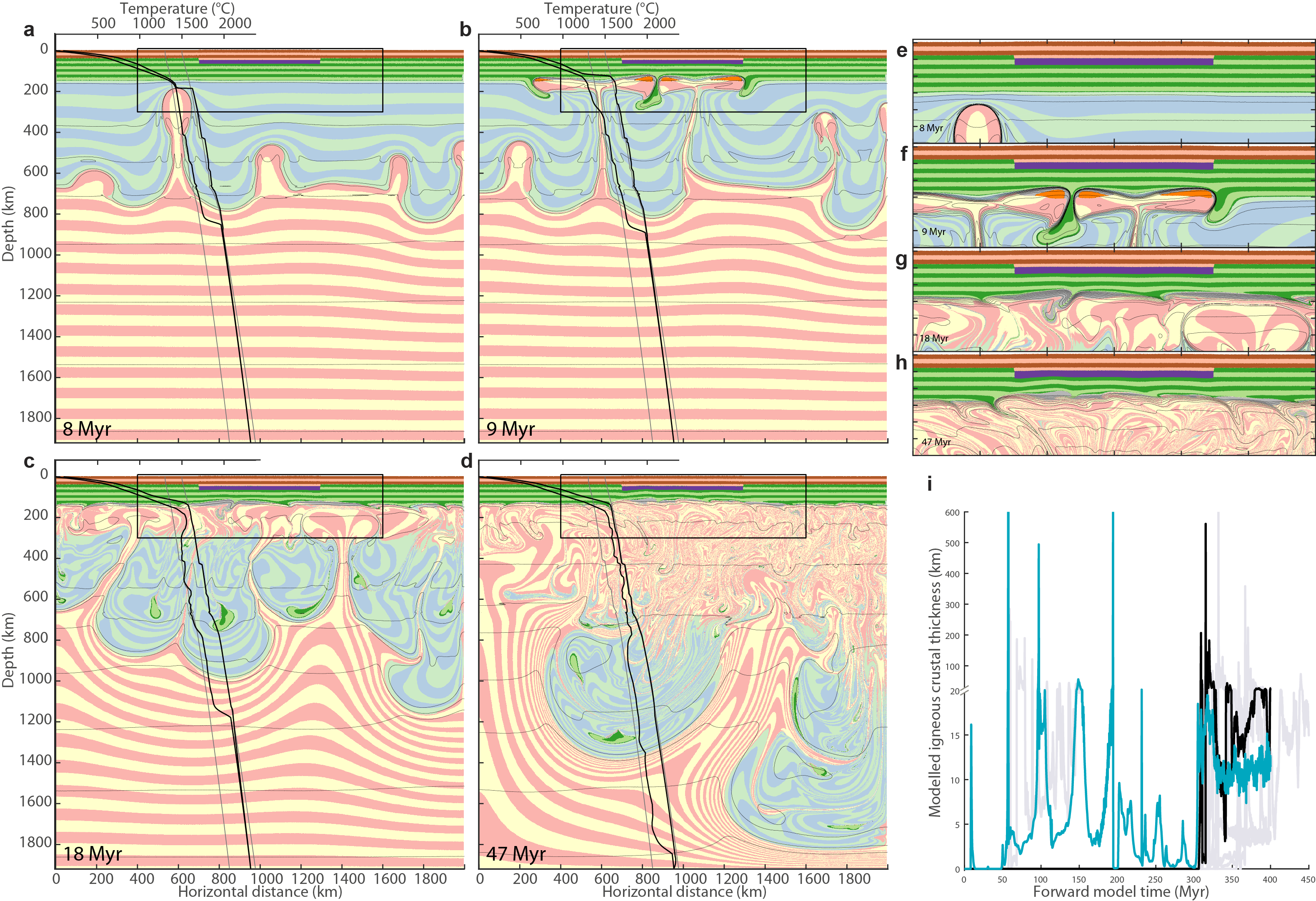


**Figure S7 | Model without a MORB graveyard at the LUMB.** The model is otherwise similar to the reference model. Colors and lines in (**a**-**d**) and the close ups in (**e**-**h**) indicate lithology, isotherms and temperature profiles in the same way as is in Fig. 1a-l. (**i**) Melt productivity of the corresponding model (a-h) is shown in blue and melt productivity of the reference model (e.g. Fig. 3) is shown in black for comparison. The melt productivity of all the other models presented in this supplement are shown in light grey. Note the broken y-axis at 20 km. This model illustrates the role of the MORB graveyard material in the reference model, where a potential temperature difference 200 °C between the upper and lower mantle is sustained for 300 Myr until rift-induced delamination destabilizes the thermal stratification. In the lack of a MORB graveyard, Rayleigh-Taylor instabilities rapidly start to form and hot lower mantle rises and melts after only 9 Myr.


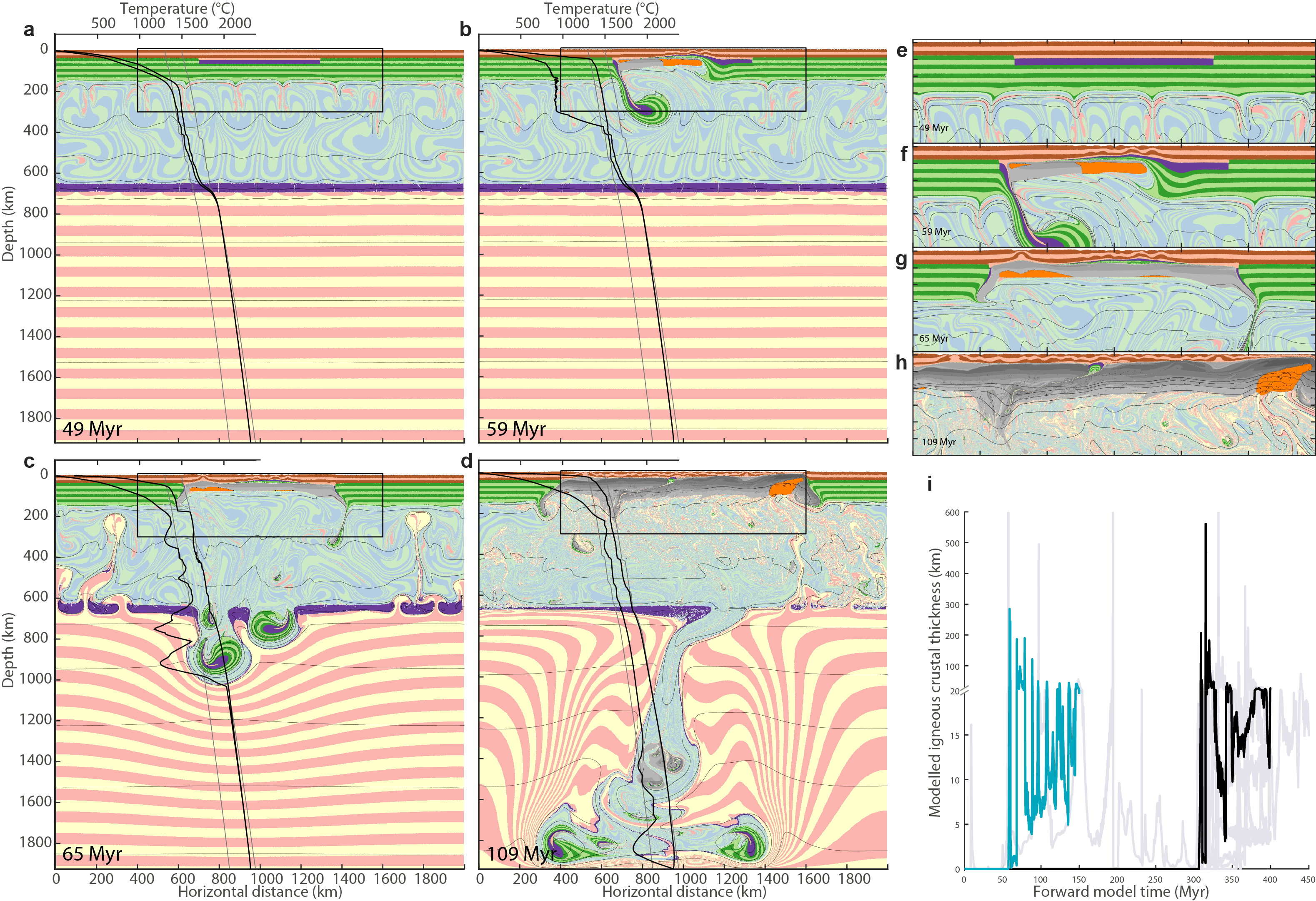


**Figure S8 | Model where extension onsets at 50 Myr.** The model is otherwise similar to the reference model. Colors and lines in (**a**-**d**) and the close ups in (**e**-**h**) indicate lithology, isotherms and temperature profiles in the same way as is in Fig. 1a-l. (**i**) Melt productivity of the corresponding model (a-h) is shown in blue and melt productivity of the reference model (e.g. Fig. 3) is shown in black for comparison. The melt productivity of all the other models presented in this supplement are shown in light grey. Note the broken y-axis at 20 km. The evolution of this model is generally similar to that of the reference model except that the chain of events, rifting, delamination and lower mantle upwelling, occur 250 Myr earlier.


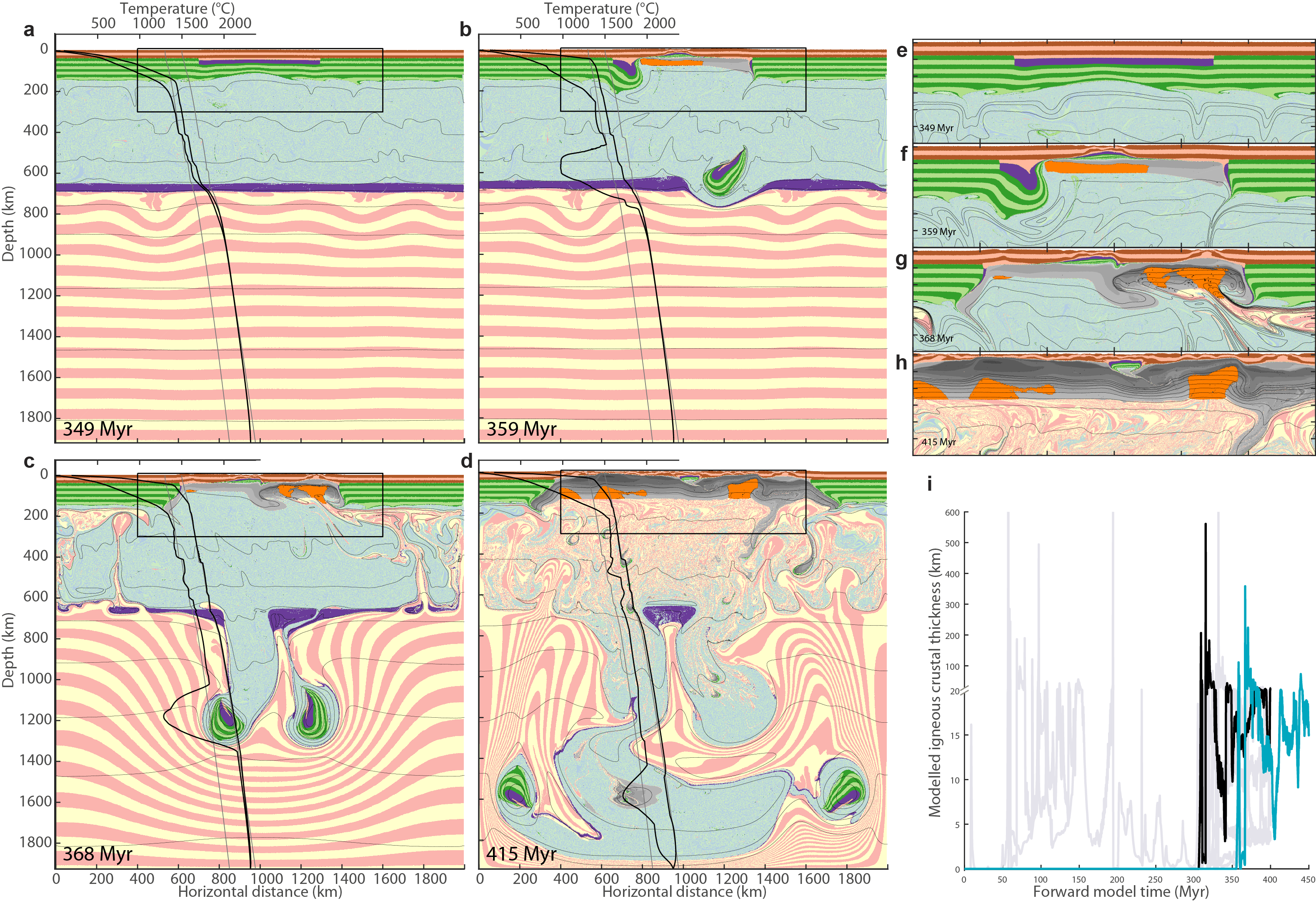


**Figure S9 | Model where extension onsets at 350 Myr.** The model is otherwise similar to the reference model. Colors and lines in (**a**-**d**) and the close ups in (**e**-**h**) indicate lithology, isotherms and temperature profiles in the same way as is in Fig. 1a-l. (**i**) Melt productivity of the corresponding model (a-h) is shown in blue and melt productivity of the reference model (e.g. Fig. 3) is shown in black for comparison. The melt productivity of all the other models presented in this supplement are shown in light grey. Note the broken y-axis at 20 km. The evolution of this model is generally similar to that of the reference model except that the chain of events, rifting, delamination and lower mantle upwelling, occur 50 Myr later.

**Table S1 | Numerical modelling parameters**

| **Description** | **Symbol** | **Value** |
| --- | --- | --- |
| **Freely deforming upper layer** |  |  |
| Viscosity | *η* | 10^21^ *Pas* |
| Yield strength | *σ_y_* | 0.1 *MPa* |
| Shear modulus | *μ* | 1 *GPa* |
| Density | *ρ* | 1 *kgm^-3^* |
| **All Lithologies** |  |  |
| Angle of internal friction | φ | 36° (linearly reduced to 0° as plastic strain approaches 0.1) |
| Cohesion | *C* | 30 *MPa* |
| Density as function of *T* and *P* | *ρ* | Calculated using Perple_X. |
| Isobaric heat capacity and entropy change | *C_p_ , dS* | Calculated using Perple_X. |
| **Crust** |  |  |
| Thermal conductivity | *k* | 2.5 *Wm*^-1^*K*^-1^ |
| Shear modulus | *μ* | 25 *GPa* |
| Radiogenic heat production | *H_r_* | 1 *μWm^-3^* |
| Activation energy^50^ | *E* | *238 kJmol^-1^* |
| Power-law exponent^50^ | *n* | *3.2* |
| Power-law constant^50^ | *A* | 2.08·10^-23^*Pa^-n^s^-1^* |
| **Mantle** |  |  |
| Shear modulus^53^ | *μ* | 80 *GPa* |
| Entropy change due to melting | *ΔS_melt_* | 300 JK^-1^kg^-1^ |
| Radiogenic heat production |  | 2.8 *pWkg^-1^ ρ* |
| *-Upper* |  |  |
| Activation energy (dislocation creep)^53^ | *E* | 540 *kJmol^-1^* |
| Power-law exponent (dislocation creep)^53^ | *n* | 3.5 |
| Power-law constant (dislocation creep)^53^ | *A* | 2.41·10^-16^*Pa^-n^s^-1^* |
| Activation volume (dislocation creep)^53^ | *V* | 15 *cm*^3^*mol*^-1^ |
| Activation energy (diffusion creep)^53^ | *E* | 300 *kJmol^-1^* |
| Power-law exponent (diffusion creep)^53^ | *n* | 1 |
| Power-law constant (diffusion creep)^53^ | *A* | 3.50·10^-10^ *Pa^-1^s^-1^* |
| Activation volume (diffusion creep)^53^ | *V* | 6 *cm*^3^*mol*^-1^ |
| *-Lower* |  |  |
| Activation energy (diffusion creep)^54^ | *E* | 200 *kJmol^-1^* |
| Power-law exponent (diffusion creep)^54^ | *n* | 1 |
| Power-law constant (diffusion creep)^54^ | *A* | 1.30·10^-16^ *Pa^-1^s^-1^* |
| Activation volume (diffusion creep)^54^ | *V* | 1.1 *cm*^3^*mol*^-1^ |
|  |  |  |

**Table S2 | Chemical composition of the 3 lithologies employed in the model.**

|  | Na_2_O | CaO | FeO | MgO | Al_2_O_3_ | SiO_2_ |
| --- | --- | --- | --- | --- | --- | --- |
| Pyrolite | 0.4082 | 3.1633 | 8.1633 | 38.878 | 3.3673 | 46.0204 |
| MORB | 2.8403 | 11.838 | 9.2049 | 7.9403 | 16.689 | 51.4875 |
| Continental crust | 4.0586 | 4.3774 | 4.7705 | 15.8309 | 15.8309 | 68.6677 |

**Supplementary reference**

74. Schubert, G., Yuen, D. A. & Turcotte, D. L. Role of phase transitions in a dynamic mantle. *Geophysical Journal of the Royal Astronomical Society* **42**, 705-735, (1975).
